# A collection of narrative practices on cultural heritage with innovative technologies and creative strategies

**DOI:** 10.12688/openreseurope.14178.1

**Published:** 2021-10-25

**Authors:** Estefanía López Salas

**Affiliations:** 1 Department of Project Design, Urbanism and Composition (DPAUC), Universidade of A Coruña, A Coruña, 15071, Spain

**Keywords:** Pilgrimage routes, Rural Europe, Cultural Heritage, Digital Storytelling, Locative Media, Multimedia narratives

## Abstract

The H2020 project rurAllure, “Promotion of rural museums and heritage sites in the vicinity of European pilgrimage routes” (2021-2023) aims to enrich pilgrims’ experiences with the creation of meaningful cultural products focused on the lesser-known heritage sites of rural areas that are not found on pilgrimage routes, but in their surroundings. One of the project goals is to create contents and narratives to be offered to pilgrims over successive days with the integration of state-of-the-art technology. This way, hidden rural heritage will be discoverable and pilgrims will have the opportunity to actively engage with rural places nearby, their local communities, identity, and culture. The latter will no longer be passive witnesses, but active participants in transnational networks of shared history and living heritage.

The rurAllure project aims to develop a new concept of mobile guide for pilgrims that will present rural heritage sites and activities of interest along with information of transportation and accommodation to help movement from and back to pilgrimage routes, as well as cohesive narratives to be consumed along the way, focused on four pilots: literary heritage on the ways to Santiago de Compostela, thermal heritage and others on the ways to Rome, ethnographic heritage on the ways to Trondheim, and natural heritage on the ways to Csíksomlyó.

To facilitate the pilots’ brainstorming in the creation of multimedia contents, we developed a review of narrative models on cultural heritage storytelling. In this paper, we present the results, a collection of 22 case studies we analyzed with a common structure, from which six distinctive groups of narrative practices emerge: sound-walks, wearable guides, context-aware games, simulations, digital exhibitions, and cultural wayfinding. All cases studies disrupt traditional notions of storytelling consumption and foster new relationships between people and places of interest that may lead to advancements in the pilgrimage context.

## Plain language summary

The present review provides a collection of best practices and actions that utilize the latest digital technologies and innovative strategies for producing meaningful narratives, mainly about cultural heritage sites. All of them are focused on the active engagement of users with particular locations of interest, landmarks, structures, objects, or intangible expressions. By doing so, their creators try to enhance tourists’ experiences while they explore or travel to places with cultural, natural, or historical significance.

This state-of-the-art collection was developed within the H2020 project rurAllure, “Promotion of rural museums and heritage sites in the vicinity of European pilgrimage routes” (2021–2023). The project is focused on attracting en-route pilgrims to hidden or lesser-known rural museums and heritage sites located near four European pilgrimage routes: Santiago de Compostela, Rome, Trondheim, and Csíksomlyó. With this collection, our aim is to develop the project in the creation of multimedia contents and multimodal narratives for people that make their way to these pilgrimage destinations based on an initial, precise approach to the latest actions in the field.

## Introduction

In the first chapter of his book, *The Mobile Story: Narrative Practices with Locative Technologies*, Jason Farman sees contemporary projects of site-specific digital storytelling as a new form of a centuries-old tradition. These projects consist of tying narratives to places that were described onsite through permanent or ephemeral inscriptions. The aim was to make some aspects of the location related to history, conflicts, religion, or architecture, to name only a few, foundational for the experience of those places ( Farman, 2014, 3–6).

To illustrate his argument, Farman uses the example of the Stations of the Cross, also known as the Way of the Cross, the Way of the Sorrows or the *Via Crucis*. This refers to an ancient practice in the form of a short-term pilgrimage in which the first Christians walked in the footsteps of Jesus, from Pontius Pilate’s palace to the Mount Calvary along the *Via Dolorosa* in Jerusalem ( Zike, 2013). The initial way comprised 14 stations where pilgrims stopped to tell through prayer and meditate about the events that happened in those places in relation to Jesus’ last days on Earth. By standing at the site where the event took place, pilgrims got an added experiential value, a deeper sense of the story, and a stronger understanding of their position within that place ( Farman, 2014, 6–7).

**Figure 1.**
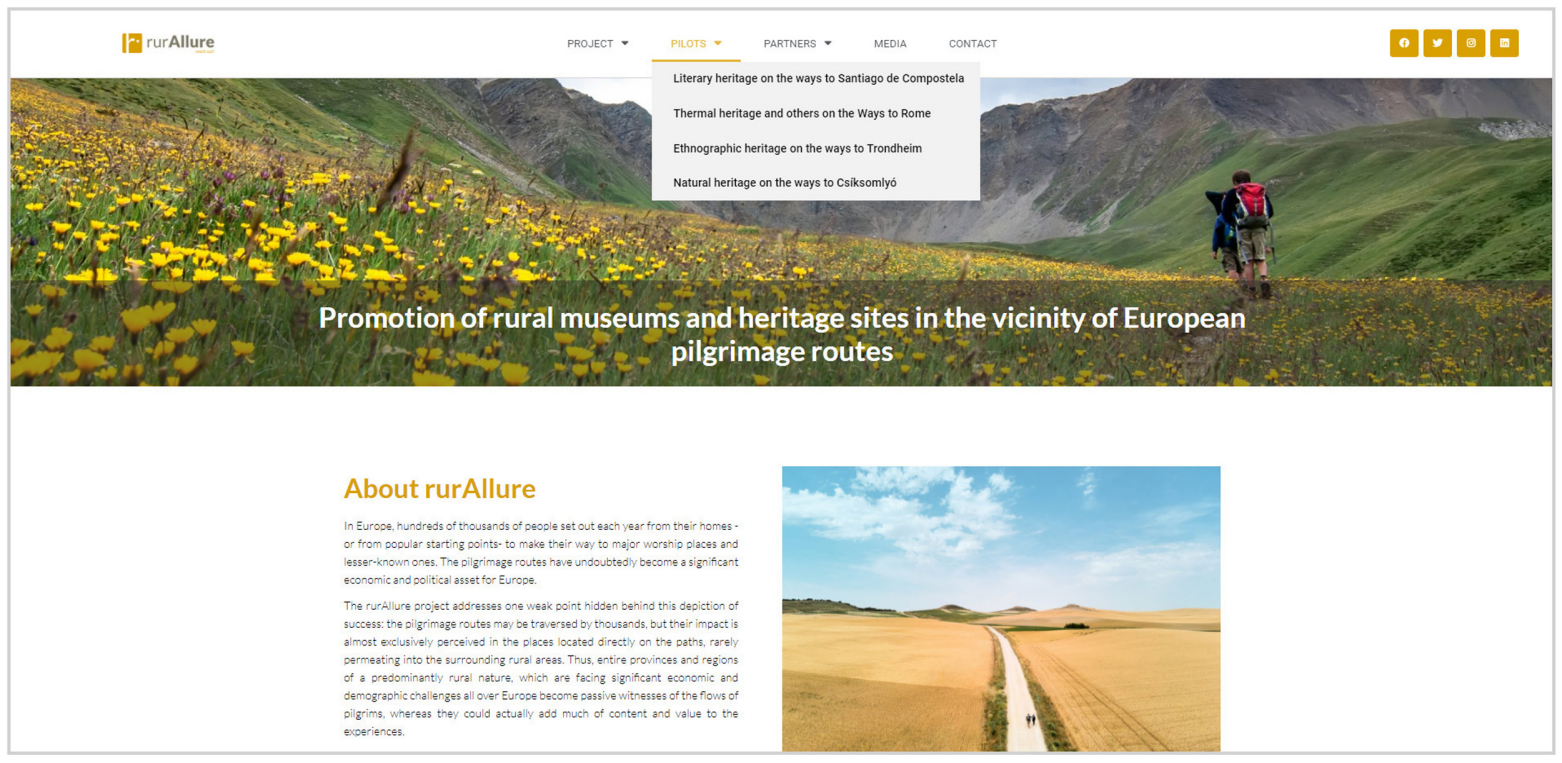
RurAllure Project website accessible at: https://rurallure.eu/.

One of the goals of the H2020 project rurAllure, “Promotion of rural museums and heritage sites in the vicinity of European pilgrimage routes” (2021–2023), is to utilize the latest technology to produce sound and cohesive narratives for four European pilgrimage routes to Santiago de Compostela (Spain), Rome (Italy), Trondheim (Norway) and Csíksomlyó (Romania). In particular, this European project is focused on engaging pilgrims with rural locations, museums, and heritage sites that are not placed along those pilgrimage routes, but in their surroundings. In addition, the selected museums and heritage sites will have their focal point at specific types of heritage, as follows: literary heritage on the ways to Santiago de Compostela, thermal heritage and others on the ways to Rome, ethnographic heritage on the ways to Trondheim, and natural heritage on the ways to Csíksomlyó ( Figure 1). With the creation of narratives that inform of the history, culture, or nature of particular locations and the people who live(d) there, rurAllure aims to enrich the pilgrims’ experiences while they walk along a route or stop at a particular place, as historical practices of site-specific storytelling embraced over centuries, but with the added value and potential of the present digital age.

With that purpose in mind, we developed a review of narrative models and previous works on cultural heritage storytelling that, in the form of a critical collection, is intended to support the brainstorming of the rurAllure pilots’ creation of multimedia contents for pilgrims. The collection of 22 cases studies we present in this paper is focused on gathering and analyzing references on how to deliver and consume cultural heritage content over successive days, as pilgrimage routes demand, and how to attract pilgrims/tourists to the lesser-known heritage sites in their vicinity. Our last goal is to offer a clear overview of the field that contributes to the development of a deeper knowledge from which new concepts of guides for pilgrims and en-route multimedia content displays could be designed or redefined for pilgrimage ways.

## Methodology

### Selection of case studies

The starting point for the selection of the 22 narrative models that the present collection comprises is their use of innovative tools for cultural heritage storytelling. Therefore, we prioritize practices and actions targeted at exploring the process of development and use of state-of-the-art technologies to deliver and consume content about nearby cultural heritage.

Secondly, case studies were selected in accordance with rurAllure project focus on particular cultural heritage categories. We gathered narrative models that have proved to enrich the communication of relevant content and engagement with literary heritage, natural heritage, thermal heritage, ethnographic heritage, cultural itineraries, and other types of tangible structures of interest (monuments, archeological sites…) or intangible cultural heritage (oral traditions, performing arts…) that are not covered by the previous categories, but our project does encompasses. We also included some case studies focused on actions that were not created and implemented for the cultural heritage domain, but that do present strategies we consider to have potential in achieving rurAllure goals.

The review and comparative investigation presented here is derived from the interpretations made via desk-research and analysis of publications (books, book chapters, articles) in the field. We also carried out a thorough search in online catalogues of best practices, award competitions, and digital agencies in research fields concerned with the technological dimensions of digital storytelling for cultural heritage (locative media, augmented reality, virtual reality, artificial intelligence, mobile phones, and smart glasses). The search was progressively refined to identify more specific aspects related to one or more of the parameters previously described by using terms such as ‘pilgrimage’, ‘pilgrimage route’, ‘cultural itinerary’, ‘literary heritage’, ‘natural heritage’, ‘location-based story’, and ‘site-specific narrative’.

In addition, delivering and consuming content about cultural heritage along pilgrimage routes poses a set of challenges such as movement through space and time (long-term displacement), predominance of outdoor activities, variety of points of interest along each route (cultural and non-cultural entities), and requirements of a mainly on-foot journey, such as a light backpack. Considering this, we also used search terms such as ‘open-air’, ‘walk’, ‘on-foot’, ‘outdoor activity’, ‘itinerary’, ‘tour’, and ‘spatial narrative’. Therefore, the present collection of selected case studies offers good examples of dealing with one or more of these challenges to enhance tourists’ experiences and knowledge, but mainly in specific cultural sites or short-term travels. We argue that all selected case studies could inspire new strategies for successive days of pilgrims’ long-term travels along pilgrimage routes, and their specific values and distinctive features.

### Levels of analysis and categorization

In the collection we present here, case studies are individually described and analyzed using the following structure: Aims, Technology, Results, Strengths and Weaknesses. The aims, briefly present the project purposes in relation to cultural heritage —or non-cultural heritage entities—, from tangible structures, monuments, landscapes, sites, objects, or collections of objects to intangible practices, representations, expressions, knowledge, and skills that communities recognize as part of their history and culture, or common interests. The second section is focused on showing the main technology/ies proposed and used to achieve the project purposes, to enable the storytelling functionalities and the engagement experience. In the third section, we summarize the project outcomes: what products, strategies, actions were created and implemented; how the applied technology changes the reception of information or media related to cultural heritage or non-cultural entities; how much the user experience benefits from them. In the last two sections, we gather information about the strengths and weaknesses of each particular project that were stressed by authors who created and implemented the narrative practice, or users who benefit from them (tourists, visitors, travelers). Here we also identify and introduce topics that we see as new opportunities or challenges that technologies bring to cultural heritage storytelling in the selected case study, and the potential applications towards their use in the pilgrimage route context.

**Figure 2.**
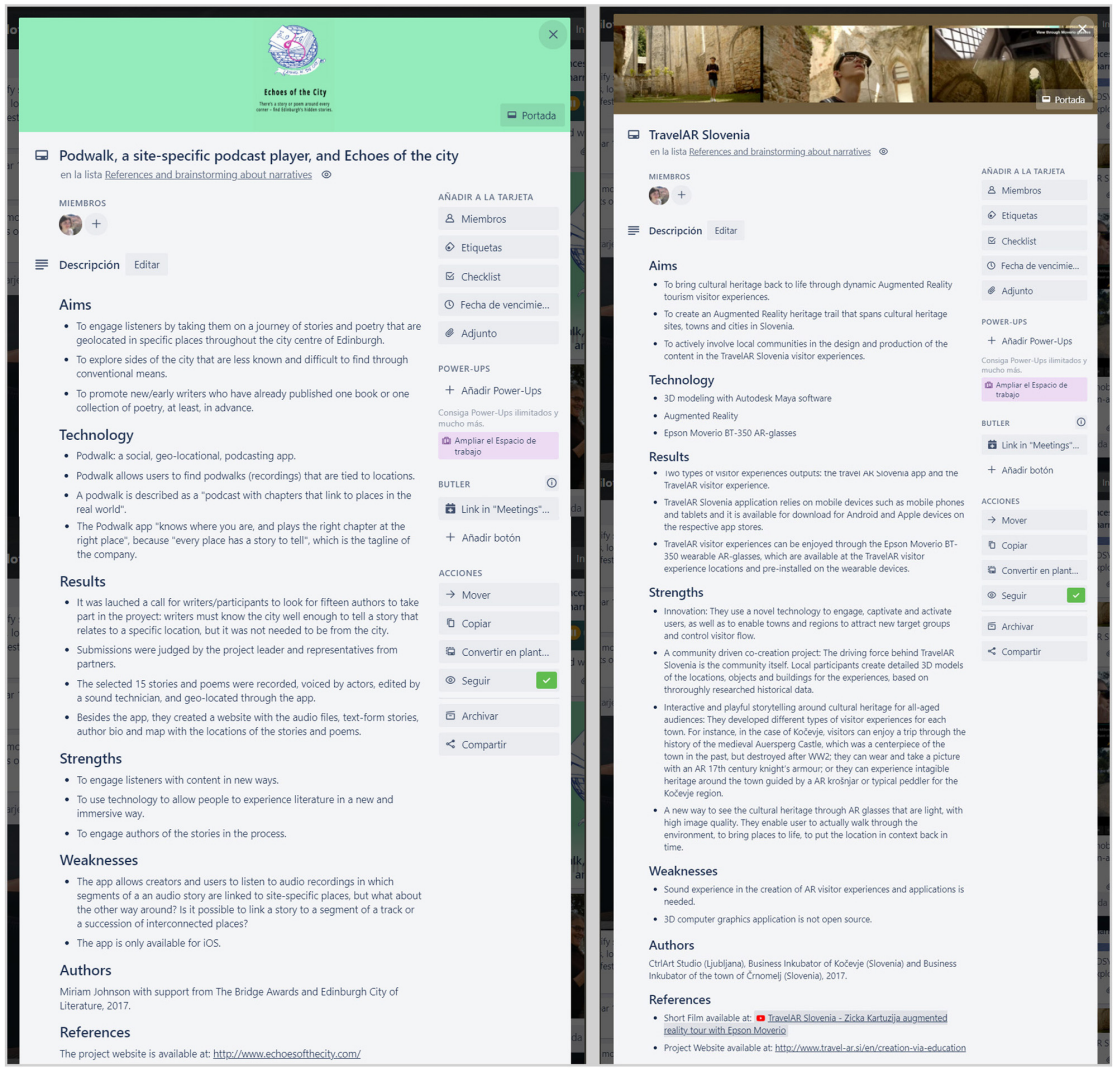
Two examples of individual cards created for two different case studies in Trello Board of rurAllure WP2.

To implement this structure of analysis, we created a workspace titled “References and brainstorming about narratives” within the Trello Board of rurAllure WP2. Then, we added a template sticky note based on the same setup to be replicated in the process of creating the collection. This way, each time we add a new sticky note from the template we begin with the exact same content: an empty space for a photo of the case study selected, five sections to be filled (Aims, Technology, Results, Strengths and Weaknesses), another space for attaching external links for further information, and a place for comments from project members. An individual card was created for each case study based on the template sticky note in Trello and we shared them with the four project pilots while the collection was being created ( Figure 2).

Once we had gathered a rich number of practices and actions, we added a new visual layer of global analysis with a twofold purpose. On the one hand, we aim to allow an easier navigation and reading through the collection of individual cards previously created in Trello. On the other hand, our goal was to enable the reader and us to extract deeper learnings on the different applied strategies and their impact, as well as to open new paths for exploration that may result from an overall examination.

**Figure 3.**
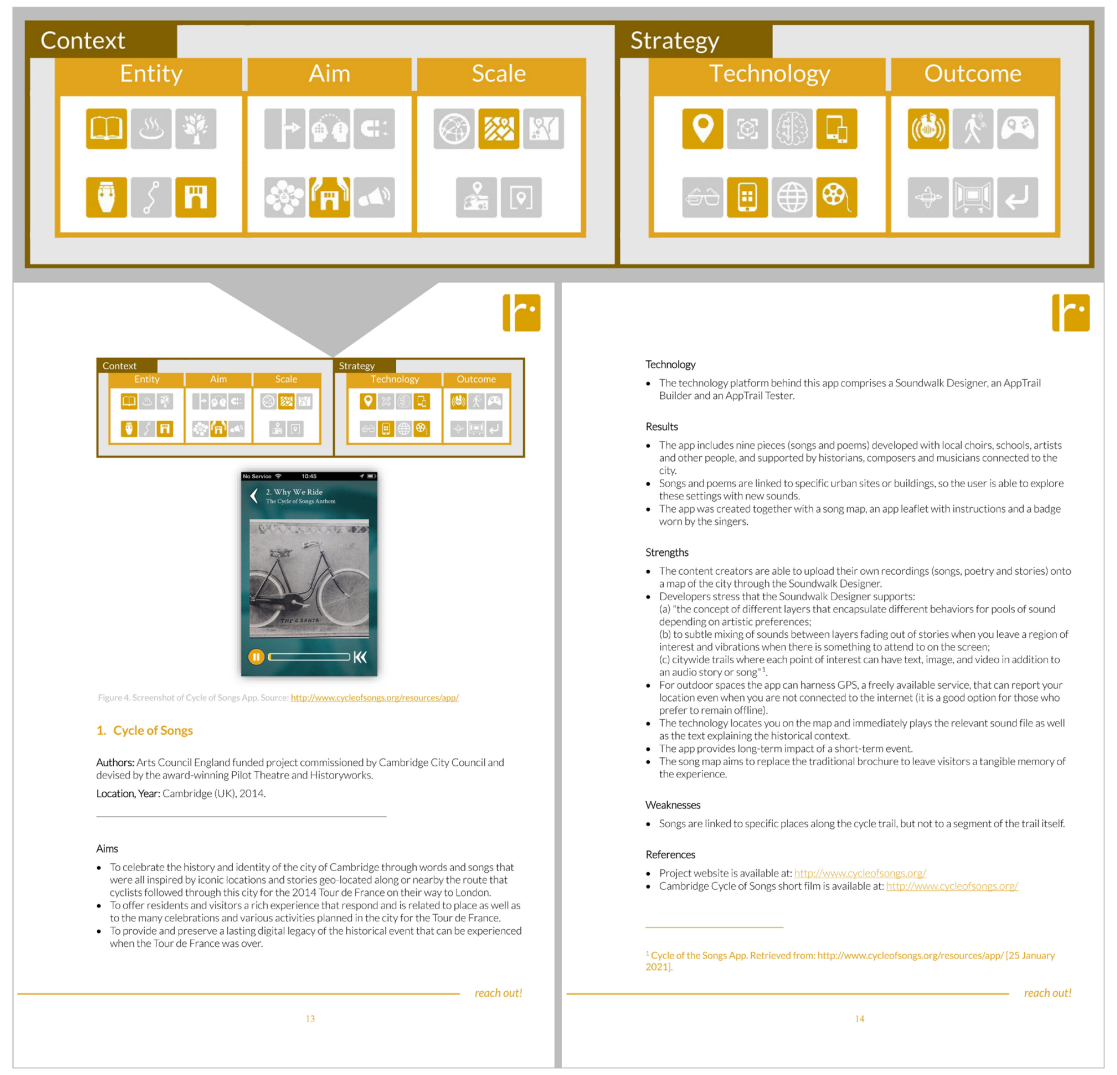
A sample of the visual representation of global analysis created for the first case study within the collection.

This global approach is based on different levels of analysis, where clear visual representation plays a key role in reading and navigation, as shown in Figure 3.

The first level describes the case study based on two categories, Context and Strategy, as it is displayed in Table 1. The item ‘Context’ represents the case study focus, purpose and scale, so it comprises three different subcategories to examine the questions of what (Entity), what for (Aim) and where (Scale). The ‘Strategy’ category represents the instruments used for the implementation of the narrative practice, project or action (tools and technology) according to the pre-set focus, aims and scale as well as the achieved outcomes. There are two different subcategories that answer the questions how (Technology) and what results (Outcomes). Each subcategory is a second level of analysis that is also further described in a third one as it is shown in Table 2, Table 3, Table 4, Table 5, and Table 6.

**Table 1.** Categories and subcategories of the first and second levels of analysis.

Category	Subcategory	Description
Context	Entity	The case study focus.
	Aim	The case study purpose.
	Scale	It points the scale of the place for and on which the project is created and implemented.
Strategy	Technology	Technology and tools used in the case study for storytelling.
	Outcome	The case study results.

**Table 2.** Possible sub-subcategories within the sub-category Entity, related to the case study focus.

Icon	Sub-subcategory	Description
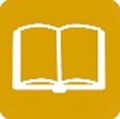	Literary heritage	The case study is focused on literary heritage, that is, authors, poets, oral traditions, written books, travel literature, travel memoirs… ( Strepetova, 2020, 18–19).
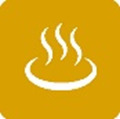	Thermal heritage	The case study is focused on different elements that constitutes thermal heritage, such as, natural heritage (the springs), building heritage (thermal structures) or intangible heritage (events, stories, people…) ( Crecente Maseda *et al*., 2018, 13–16)
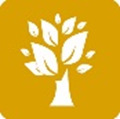	Natural heritage	The case study is focused on natural sites of value from the point of view of science, conservation or natural beauty, such as, natural areas, zoos, aquaria and botanical gardens, natural habitat, marine ecosystems, sanctuaries, reservoirs etc. ( UNESCO, 2019, 19).
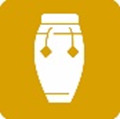	Ethnographic heritage	The case study is focused on manifestations, knowledge, customs and expressions of tangible or intangible traditional culture that define common features of different groups within a community ( Law 13/1985, 20347-20348; UNESCO, 2018, 12).
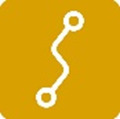	Cultural itineraries	The case study is focused on a physical route crossing one or two more countries or regions, organized around themes with historical, artistic or social interest, taking different forms according to the identity of each site or area ( Berti, 2015, 14).
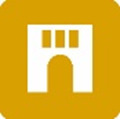	Others	The case study is focused on another type of cultural heritage that does not belong to any of the previous categories, or on non-cultural heritage entities.

**Table 3.** Possible sub-subcategories within the subcategory Aim, related to the case study purpose.

Icon	Sub-subcategory	Description
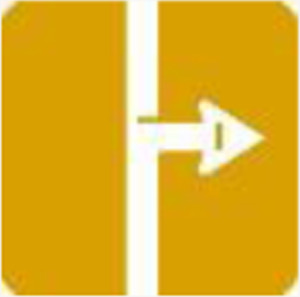	To activate	The case study aims to promote and activate lesser-known or unknown cultural heritage, in particular those off the beaten track, or non-cultural heritage entities.
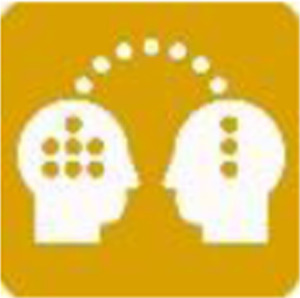	To educate	The case study aims to enhance public knowledge about cultural heritage or non-cultural heritage entities.
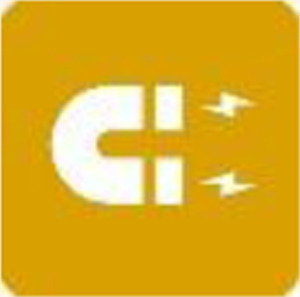	To engage	The case study aims to create community engagement with cultural heritage or non-cultural heritage entities.
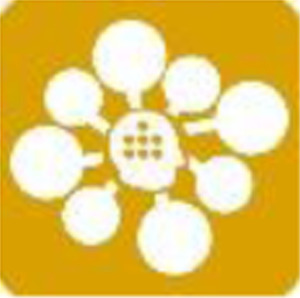	To enrich	The case study aims to enrich on-site experience of cultural heritage or non-cultural heritage entities, through more diversified presentation, interpretation and interaction that enables new approaches to and connections between heritage and the public.
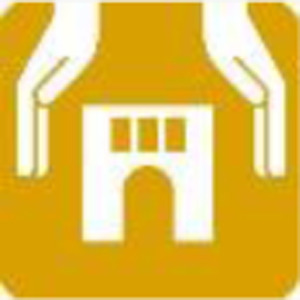	To preserve	The case study aims to digitally preserve the legacy of historical events, traditions, or any of humankind's fragile or at-risk cultural heritage for future generations.
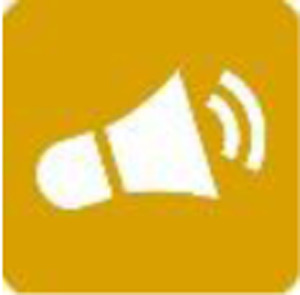	To raise awareness	The case study aims to increase public understanding of the importance of cultural heritage as a common wealth or certain non-cultural heritage entities.

**Table 4.** Possible sub-subcategories within the subcategory Scale, related to the place for and on which the case study is created and implemented.

Icon	Sub-subcategory	Description
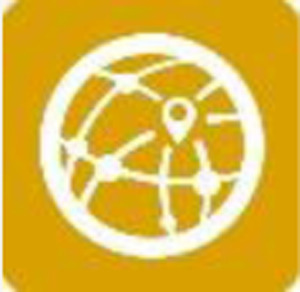	Cross-border route	The case study is created and implemented for a physical pathway that crossed one or more urban or rural areas within the same or different country.
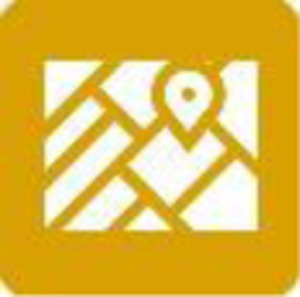	Urban itinerary	The case study is created and implemented for the space of a city, one or more neighborhoods, or a specific urban itinerary along certain streets of a city.
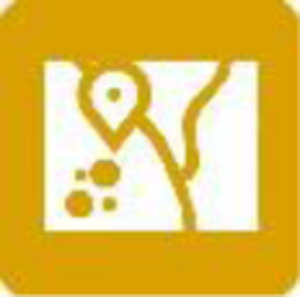	Open-air walk	The case study is created and implemented for an open-air area attached to an urban or rural context, but outside the consolidated tissue, such as, a park, a coastline.
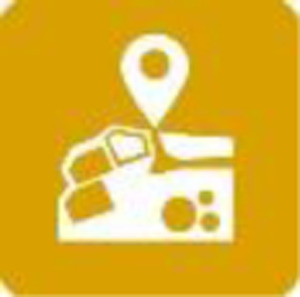	Site of interest	The case study is created and implemented for a particular area with distinctive features, such as an architectural complex, an archaeological site, or any standing structure along with their surrounding landscape, natural environment and geographical setting ( ICOMOS, 2008, 2).
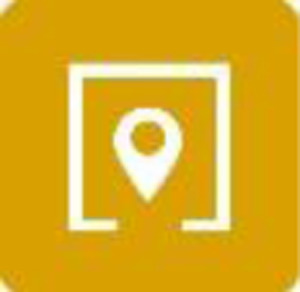	Building	The case study is created and implemented for one particular building, mainly for its inner space.

**Table 5.** Possible sub-subcategories within the subcategory Technology, related to the technology and tools used in the case study for narrative practices.

Icon	Sub-subcategory	Description
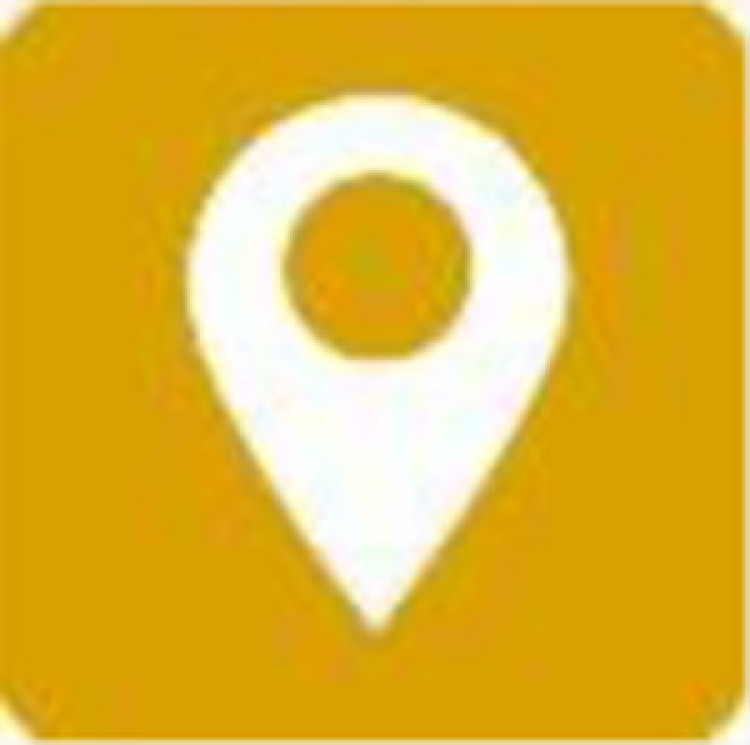	Locative media	The case study makes use of GPS to bound content to site-specific locations ( San Cornelio & Ardévol, 2011, 313–314).
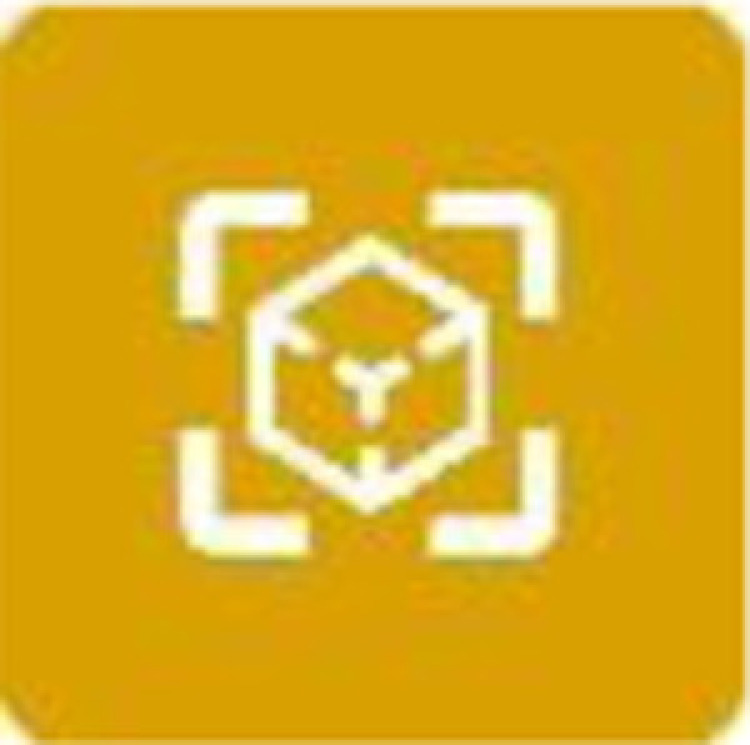	AR/VR/MR	The case study uses augmented reality (AR), virtual reality (VR) or mixed reality (MR) to superimpose 2D or 3D computer-generated data and information or virtual objects as if they coexist in the real world (AR, MR), and to allow users to visualize and interact with heritage artifacts in more intuitive, direct and appealing ways, or to create a simulated environment, detached from the reality (VR) ( Nofal *et al*., 2018).
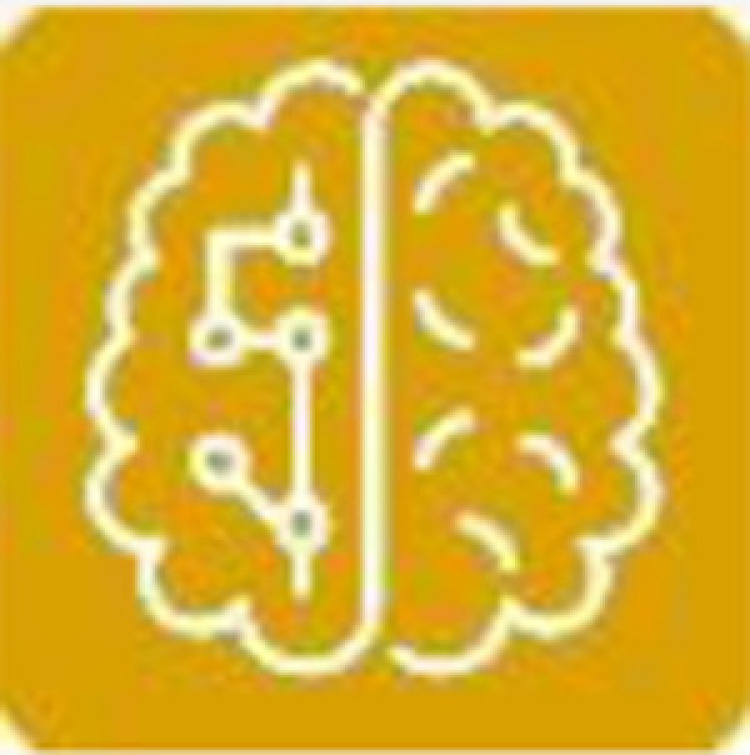	Artificial intelligence	The case study collects data on the user behavior and processes it with artificial intelligence tools, to interact with the user, or to return personalized information.
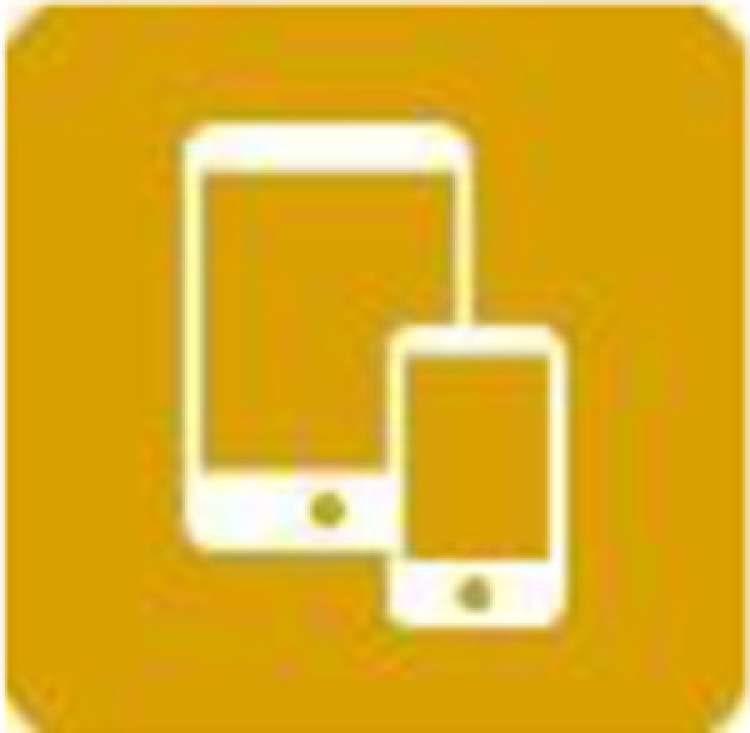	Mobile device	The case study makes use of mobile devices such as smartphones or tablets.
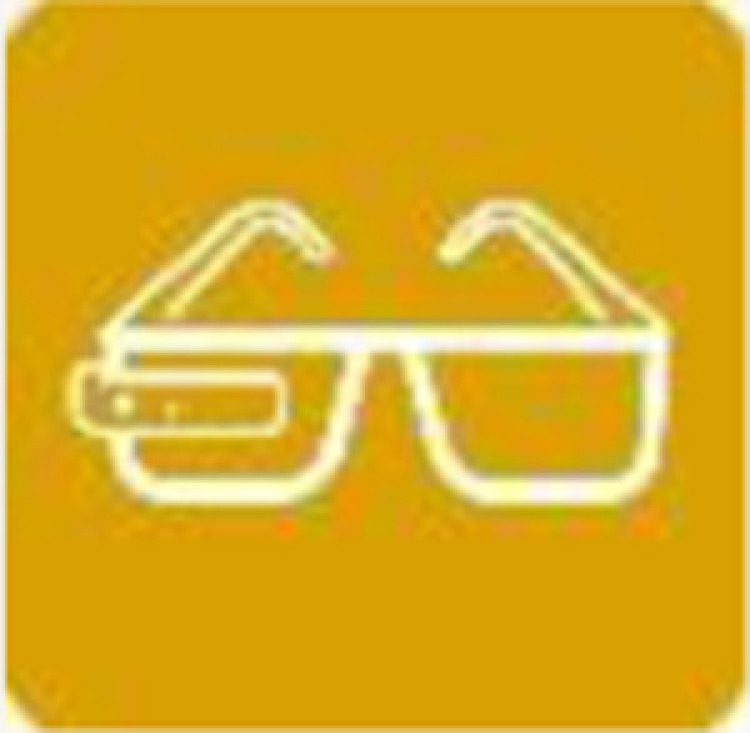	Smart glasses	The case study makes use of wearable smart glasses.
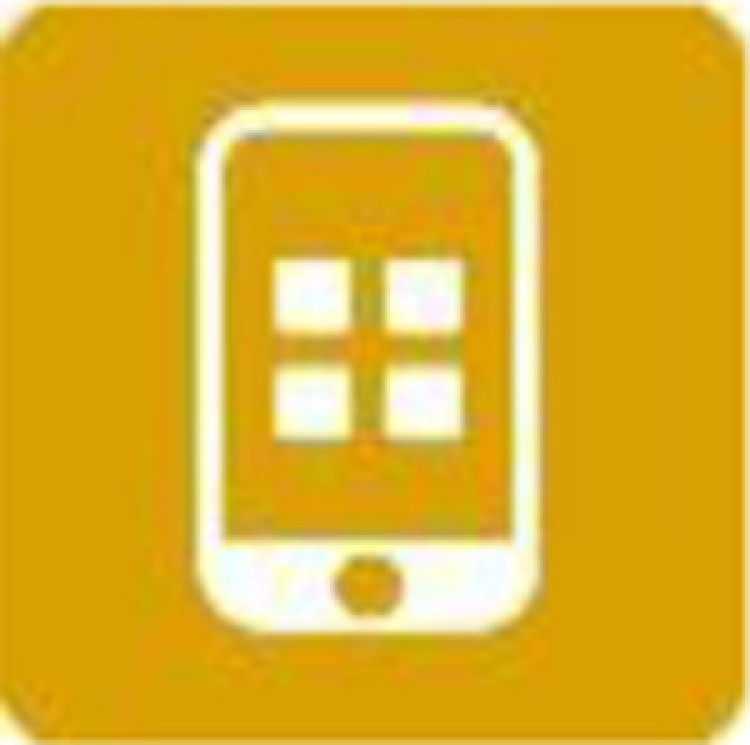	Mobile app	The case study makes use of a software designed to run on smartphones and other mobile devices.
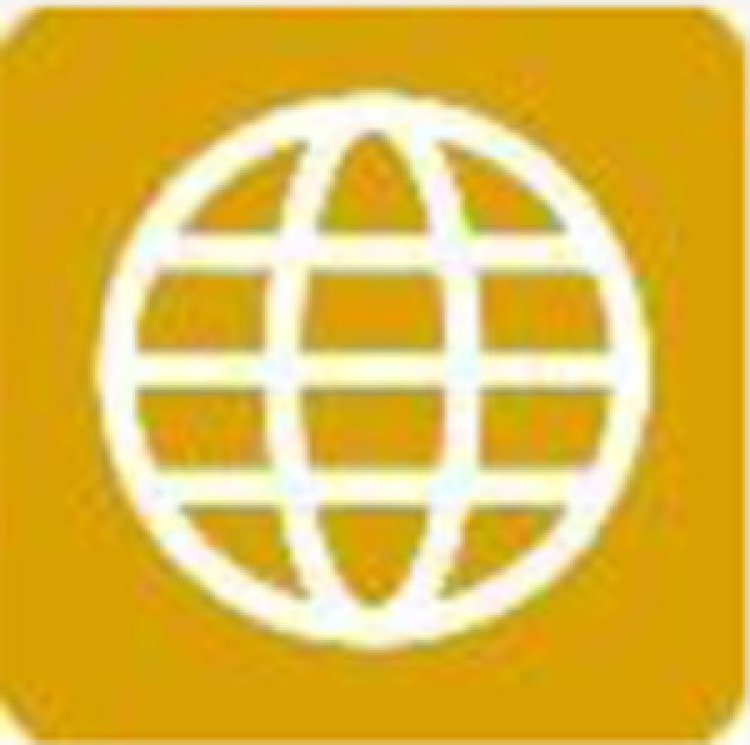	Web application	The case study makes use of a software designed to run inside a web browser.
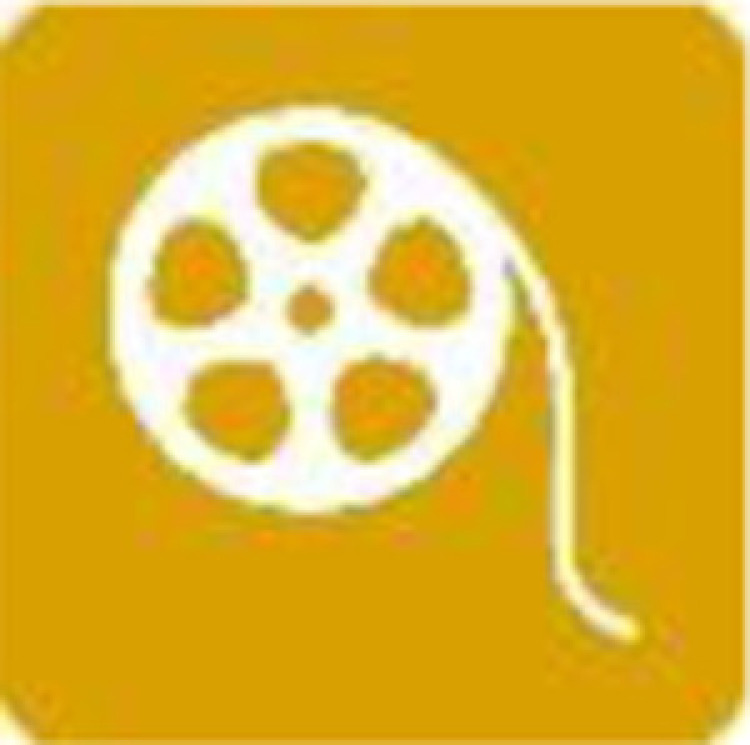	Analogue tangibles	The case study makes use of non-digital tools.

**Table 6.** Possible sub-subcategories within the subcategory Outcome, related to the case study results.

Icon	Sub-subcategory	Description
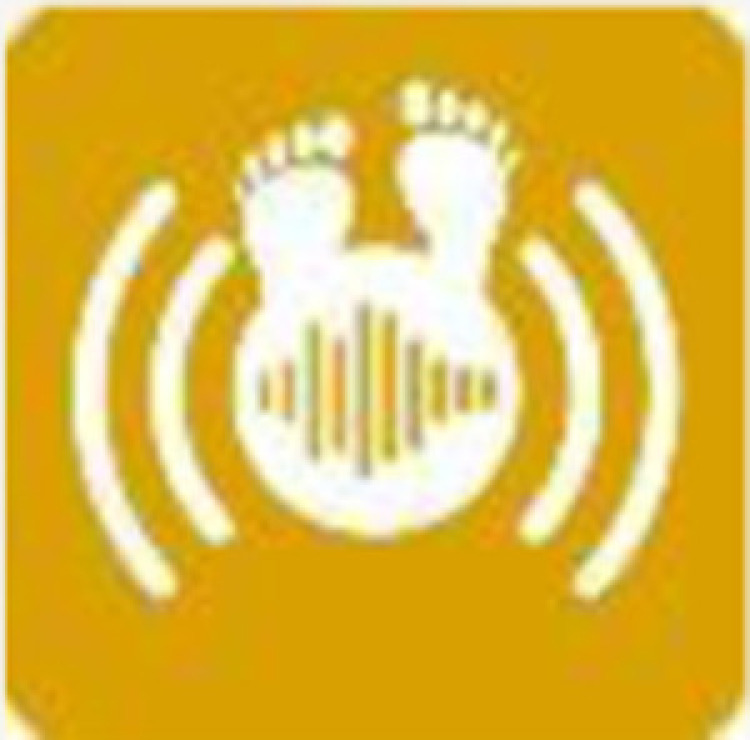	Sound-walks	The case study results in audio narratives along with or without images/video that mainly makes use of sound and/or sight to deliver content.
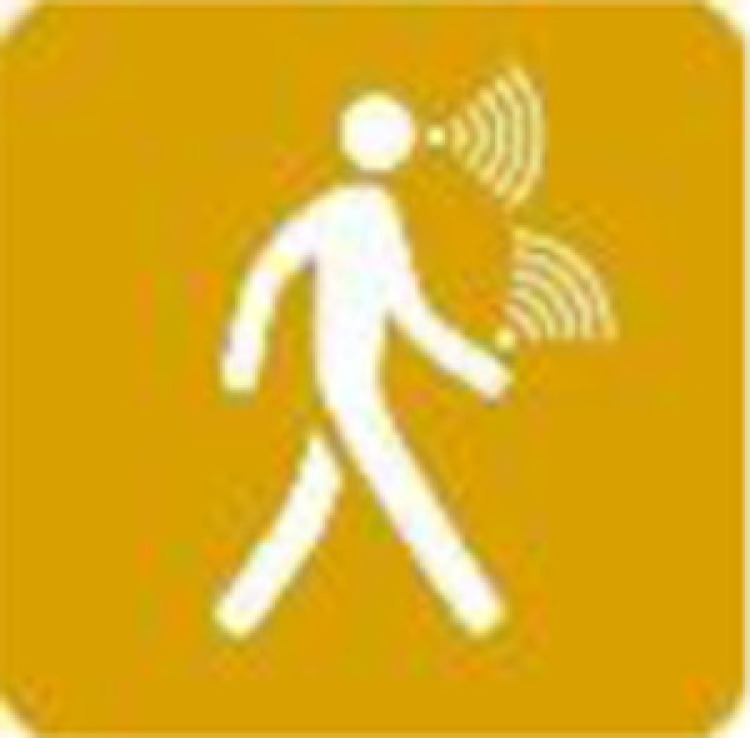	Wearable guides	The case study results in a noninvasive, autonomous and multi-sensorial integral guide that aggregates digital data to improve user experience.
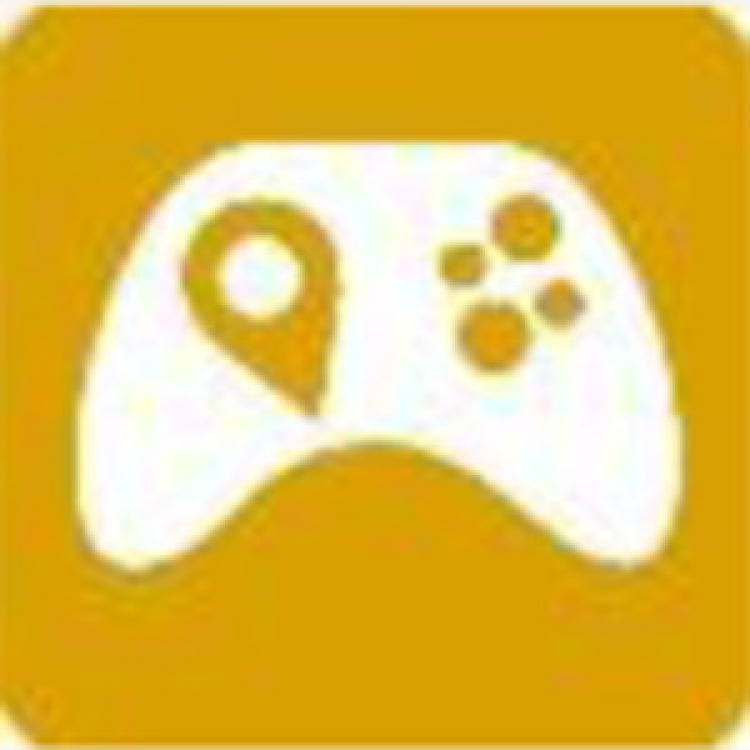	Context-aware games	The case study results in games that invite users to become a player that may interact with others in a location-based experience ( Bunting, 2014, 161–162).
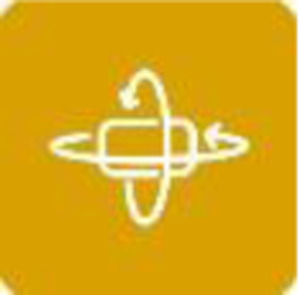	Simulations	The case study results in recreations of former historical realities that make visible the past and hidden stories attached to events, objects, landscapes, buildings.
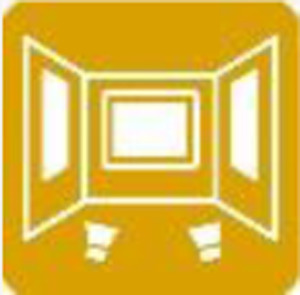	Digital exhibitions	The case study results in a digital exhibition or a set of digital exhibitions that add experiential layers of culture to physical space.
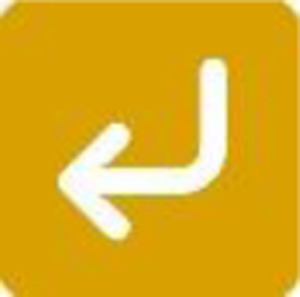	Cultural wayfinding	The project results in a wayfinding system that helps people to navigate from place to place with graphic communication, visual clues in the built environment, audible communication, tactile elements, and aggregates or collects cultural data on the way.

## Case studies

Next we present each case study, on an individual basis, but within six distinctive main groups of narrative practices that emerge from our global examination: sound-walks, wearable guides, context-aware games, simulations, digital exhibitions and cultural wayfinding. These six groups match the same number of possible outcomes that the latest applied technologies and strategies on cultural heritage narratives we review may result in, as the previous tables show.

### Sound-walks

Within the first group, sound-walks, we analyze a total of eight case studies that have in common their interest in the creation of auditory narratives to be consumed while the listener (tourist) is standing at one specific location or wandering from one place to another. These case studies are grounded within “Jeremy Hight’s” thoughts and theories around “locative narrative”, which he defines as “a new paradigm in publication and an extension of the possibility of new media narratives to move narrative from the printed page and literary journals to alternative spaces and new possibilities of dissemination, audience and community” ( Hight, 2010, 322). Hight remarks that places have a voice, and when this voice is made discoverable, places may become a new bookshelf, as “history, architecture, archaeology and other hard facts and data of a place and its present and past could be placed not in distant books and journals, but where they took place” ( Hight, 2010, 327).

All selected cases studies are locative narratives, in the form of audio files triggered by proximity to specific GPS coordinates through the use of mobile devices, such as smart phones or tablets. In addition, like in the case of the oral narrative tradition, sound-walks are heavily aligned with the visual as the user is both a listener and an observer, but with an emphasis on sound. This promotes a shift in the current creation and consumption of multimedia content that is still clearly focused on the visual sense ( Barber, 2014, 98). In that way, audio stories are connected to a physical place or a succession of places, from which they uncover a hidden story and, by doing so, they are directly interwoven into the experience of that place ( Barber, 2014, 96). Sound-walks become an added layer of intangible knowledge content that allows listeners to actually read a place and enrich physical experience ( Pettoello, 2016, 10.4).

The first case study we approach is called the Cycle of Songs. It was created to celebrate the history and identity of the city of Cambridge through words and songs that were all inspired by iconic locations and stories geo-located along or nearby a specific route: the one that cyclists followed through this city for the 2014 Tour de France on their way to London. They aimed to offer residents and visitors a rich experience that responds and is related to place and to the many celebrations and activities planned in the city for the Tour de France. In addition, another goal was to provide and preserve a lasting digital legacy of the historical event that could be experienced when the Tour de France was over.

Funded by the Arts Council England, Cycle of Songs is a collaboration between the Cambridge City Council, the Pilot Theatre and Historyworks. Using an AppTrails technology designed by the software service company Calvium, they developed the Cycle of Songs app, which includes nine pieces of songs and audio poems. These were performed by local choirs, schools, and artists, and curated by historians, composers, and musicians from the city. All songs and poems are connected to specific urban sites or buildings, so app users are able to explore and discover locations of historic and/or cultural interest through the added layer of the aural senses. To make this sound-walk even richer, they created a sound map featuring audio content on location, an app leaflet with instructions and a badge, all of which, as analogue tools, leave participants with a tangible memory of the experience.

The strategy of this case study is based on the use of a Soundwalk Designer supported by Calvium that allowed the content creators to upload their own recordings (music, poetry or narratives) onto a map of the city of Cambridge. App developers highlight that the Soundwalk Designer tool supports:

(1) *the concept of different layers that encapsulate different behaviors for pools of sound depending on artistic preferences;*
(b) *the subtle mixing of sounds between layers fading out of stories when you leave a region of interest and vibrations when there is something to attend to on the screen;*
(c) *citywide trails where each point of interest can have text, image, and video in addition to an audio story or song*. ^1^

Cycle of Songs uses GPS technology to locate participants on the map embedded in their mobile telephones and immediately play the sound file that is associated with the specific location they are walking through, whether they are connected to the internet or not. Each song or poem is tied to one urban location and all of them are linked by the city route that cyclists followed during the 2014 Tour de France. Sound narratives of Cycle of Songs are site-specific and they clearly echo a plethora of mobile-phoned-based audio tours that include locative narratives about the area surrounding the particular tour. We argue that the distinctive feature of this case study is the context, focused on the experience and preservation of a historical cycle tour that took place in a specific urban setting. For that reason, we wonder if onsite experience could also be enhanced through audio narratives that were prepared to be listened to along the space in-between two locations rather than by triggering one hot spot through proximity as it happens in this case, as a sort of path narrative. We argue that this could be a line for exploration in rurAllure project.

Our second selected case study is Echoes of the City, created by Miriam Johnson with support from The Bridge Awards and Edinburgh City of Literature. Johnson’s desire was to give a voice both to new or early writers, who have already published one book or one collection of poetry, and to the lesser-known sides of the city of Edinburgh, where stories were rich, but still untold.

To achieve that goal, she made use of a social, geo-locational, podcasting app, called Podwalk, which allowed users to find recordings tied to specific locations. Each Podwalk was said to be “a podcast with chapters that link to places in the real world”. ^2^ The iOS application was able to know where the user was standing and played the chapter corresponding to that specific place. This is clearly a new form of literature that takes advantage of the possibility of new media narratives, as Hight points out. Moreover, it clearly fits the second avenue that Hight recognizes when he questioned where locative narrative may move literature to, as Echoes of the City does not place pre-existing literature by geo-location, but creates new literature that is composed with/for a particular place and experienced/read in the physical world itself ( Hight, 2010, 323).

A total of 15 authors took part in this project. They were selected through a call for participants in which they demonstrated they know the city well enough to tell a story that relates to a specific location, without needing to be a local writer. ^3^ Then, the 15 stories were recorded by actors, edited by a sound technician, and geo-located through the Podwalk app. In addition, they made all the new stories or poems and their audio files, along with the text-form narratives and locations, available on the project website.

We argue that Echoes of the City presents two main strengths: to engage authors of the stories in the process of creating the auditory narratives, and to provide listeners with a new form of literature connected to place that is prepared to be experienced in an immersive way, giving a new voice to certain urban settings that may otherwise remain unexplored.

The next example is the case of the Walk the Wall Athens App, implemented by the Society for the Study of Ancient Topography (Dipylon) together with Fluidmedia and the City of Athens in 2018. Their aim was to offer tourists a tool to explore and discover the hidden world of the ancient walls of Athens. For that purpose, they developed a GPS-enabled application, which includes an interactive map with 35 points of interest. These were selected from 180 archaeological sites where remains of that cultural heritage entity have been studied up to now.

As Dipylon highlights, the Walk the Wall Athens App provides users with: access to information about the walls on the go, accurate coordinates to easily spot the archaeological remains, and a series of audio narratives featuring audio content to learn about each point of interest (POI) along with rich written and visual material, a historical timeline overview, and a glossary of archaeological terms. ^4^

The app is said to be easy and simple to use, and to navigate from one POI to another by checking the interactive map. While visitors walk, they are able to get information only from the walls or from a particular featured location along a pre-set tour. However, it seems that the app does not include audio announcements or device vibrations to let participants know when they approach or arrive at a POI. This additional feature may enhance onsite exploration by means of noninvasive digital interfaces, where aural narratives are unbound from the mobile device screen, and participants’ attention is not displaced from the cultural asset.

In the case of the Walk with Me App, the project focus was not a building entity, but a series of poems and prose created by writer Anna Maria Murphy. These are based on tales, folklore and local rumors she heard in person while she travelled throughout Cornwall (UK). By using locative media, she aims to make accessible and keep alive all these stories, in a way they remain connected to the places and people she collected them from. She also tries to engage with locals and visitors in a creative way, through a sort of live theatre turned into an artistic digital experience ( Bond & Rickard, 2013).

The Walk with Me App offers six guided tours around six Cornish locations: Perranporth, Mevagissey, Newquay, Great Flat Lode, Bodmin and Helston. Each of them contains a collection of sound local stories, film snippets and photos along with bespoke route maps for each walk annotated with illustrations and icons. ^5^ Once the participants start the app and plug in their headphones, they can wander freely, discovering stories and music that pop up in certain areas marked on the map, as if they were walking with the poet that created the stories.

The Walk with Me App allows people to listen to stories exactly where they were first collected as they are based on real walks. These were carried out in the less travelled roads of Cornwall, where writer Maria Murphy invited guests to walk with her, meet people along the way and collected stories that, later on, were exaggerated to create entertaining, surprising and often moving narratives ( Bond & Rickard, 2013). These stories were designed to engage participants with the immediate world, but also to articulate that physical world. Rita Raley calls this practice a narrative environment, a kind of experiential storytelling in-between the real and the fictional, with three structural features and modes of engagement: experience, movement and environment ( Raley, 2010, 302–303). Similarly to the Walk the Wall Athens App, the Walk with me App uses GPS tracking to trigger audio files when the participant approaches a specific location, but checking the map while you walk is still needed in order to know what places in the surroundings have sound stories attached.

The **Clifton Suspension Bridge App** is another example of the use of locative media and digital narratives to educate and increase public understanding of the importance of cultural heritage as a common wealth. Developed by the University of the West of England, the Clifton Suspension Bridge Trust and Calvium in 2014, it aims to offer information about a particular heritage building, the Clifton Suspension Bridge in Bristol (UK), even when the visitor center is closed. ^6^

Based on an AppTrail software that connects locals and visitors to the place where they are standing, the Clifton Suspension Bridge App includes multimedia content (audio, image and text) to tell the history and unknown stories of the monument, whether you are onsite or not. ^7^ In this particular case study, using locative narratives allowed the creators to overcome the fact that tangible boards to display on location any kind of information about the bridge were not permitted, as they deal with a listed building. Therefore, the strategy not only enriches the visitors experience with an added layer of digital content to the physical structure and its context, but it also enables creators to help with the protection and respect of the heritage site, which is an added value of the digital realm for the interpretation, presentation and conservation of the past.

Our sixth case study is the **Cultural Roadmapp (Ireland Clare)**. The first distinctive feature of this project is the scale. While previous sound-walks were designed for and took place on a small scale (one building or site of interest), or medium scale (a city/urban itinerary), Cultural Roadmapp (Ireland Clare) is developed for a cultural itinerary that crosses different regions within the same country, that is, a large scale of action. In addition, it is the first example focused on a cultural itinerary or a physical route crossing one or two more regions, organized around themes with historical, artistic or social interest, as we previously described. In this case, the cultural itinerary is the Wild Atlantic Way in Ireland. It is a road trip along the Irish west coast, from the Inishowen Peninsula in Donegal to Kinsale in Cork. ^8^

In order to enrich the travel experience along Ireland’s Wild Atlantic Way, Deborah Schull and Leah Bernini Cronin envisioned and created the first road trip app in a four-part series, called Ireland Clare. ^9^ Launched in 2017, the app is focused on making the richness and complexity of local culture and heritage visible through stories that move with en-route motorists within the Clare county. It includes 11 audio tour stops or short stories to be listened to at particular locations along the way. ^10^ They intertwine interviews with elders, cultural experts, and local important people that are automatically displayed by a mobile device when a motorist approaches a stop from any direction. Stories are rooted in authentic culture and history as they are directly gathered from local communities. This not only guarantees a genuine and immersive experience, but it is also a powerful tool for the documentation and preservation of at-risk intangible heritage.

Another strength of the application is that no user’s visual attention on the mobile screen is demanded as it has a fully hands-free functionality. This way, visual senses are not compromised and the user is free to focus on the surrounding landscape. In addition, the creators of this app note that their tour:

*…help[s] to redistribute the tourist footprint as they track routes between cities or towns—helping to disperse tourist traffic (and dollars) toward the less-served hospitality-related businesses and hidden gems and encouraging visitors to explore just a little further, look a little deeper, and have even more memorable experiences as a result* ( Schull, 2020).

In this sense, this case study proposes the use of locative narratives to move tourists off the beaten track and, by doing so, activates the lesser-known or hidden cultural heritage, as the rurAllure project envisions for pilgrimage routes and their rural surroundings.

Another example of locative media with a large scale is DIY Tourguide. This aims to provide self-driving travelers along Central Australia with sound insights into the area they are driving through, with audio recordings from locals that talk about history, ancient Aboriginal culture, natural landmarks and wildlife fauna. DIY Tourguide offers both a digital product and analogue tangibles in the form of an audio tour app with geotagged tracks, and audio recordings in CDs or downloadable MP3 audio files.

So far this team have created two tours along the most popular routes through Central Australia. Both provide users with local insights and stories from Australian characters, locals, and experts. The first route goes from Alice Springs to Uluru (460 km). It is a desert area, but there are plenty of secret stories to be discovered about Aboriginal people, cattle owners, national parks, the landscape, flora and fauna. The second route, West MacDonnell Ranges, is shorter in distance, 132 km. The starting point is also Alice Springs, but it ends at Glen Helen Gorge. It also crosses a rich natural environment with various aspects to explore.

For the first route they created an audio tour that is divided into two parts each comprising nine tracks or sound-walks (drives) of which short samples are offered on the project website. ^11^ The second audio tour includes twelve tracks or audio stories. All stories are GPS-triggered, so the audio narrative is played when the user is driving the car along a specific area, even if they are not connected to the internet. This fact introduces a slight difference from the previous case studies that may lead to new experiential results, as stories are not ‘read’ when the user is standing at a particular location, but crossing certain areas over a long journey. Audio stories are created to give drivers freedom enough to stop where and when they want, but with new knowledge about the culture, history and nature relating to the surroundings.

The very last case study included in the group of sound-walks is VoiceMap. Founded by Iain Manley, it is a powerful web platform and mobile application in the field of audio narratives for urban tourists worldwide. It was born with a twofold purpose. Manley aimed to provide local people with a medium to share stories that are not focused on the most visited places within a touristic destination, “but rather spots they have a personal affinity to” ( Webintravel, 2017). In this way, he tried to give a voice to the local community, who not only have a deeper connection to a place than a tour company, but also a much more personal understanding and knowledge that may lead to more meaningful experiences created for others ( Manley, 2015). He also tried to offer tourists/listeners the possibility of discovering a place through the voice and immersive story of someone who is from that place ( Webintravel, 2017).

VoiceMap makes use of both a software designed to run on smartphones and other mobile devices and inside a web browser. The latter is a publishing web platform for GPS audio tours that anyone can use to publish their own audio narrative using eight simple steps, as follows: (1) logging into the platform; (2) mapping a one hour route, which returns a word count for the story according to walking times; (3) writing the script of an immersive story and sending it to the platform editors; (4) receiving the editor’s response and help with the location-aware audio and publishing tool; (5) testing the tour onsite through a trial audio recording and editing before the final recording; (6) recording the audio story; (7) uploading the audio story to be checked by platform editors and sound engineers; and finally, (8) publishing the tour by adding a title, cover photo and description. ^12^ Prior to this process, creators are asked to choose between six different types of tours according to the means of transportation: walking tour or indoor tour, driving tour, train tour, cycling tour and boat or ferry tour.

All tours created within the platform are later accessible for tourists or anyone interested in the exploration of a place at their own pace through a Walking Tour App. One of the strengths of VoiceMap is to involve in the project both the creators and the participants. They provide the former with an easy-to-use platform to tell hidden stories with instant updates, integrated support and cost-effective results that lead to plenty of possible experiences to be lived by the latter. However, as Manley notes, the audio quality of these tours may not be as good as of those offered by storytelling sound experts, although the whole process of the creation and publishing is checked by platform editors ( Webintravel, 2017). Manley created a medium to compose and deliver native mobile narratives that emphasizes the exploration of places and engages visitors with their immediate environment through the voice of people who know those places better, which are mainly urban so far. This medium is open to anyone, as he built a framework rather than a project-specific solution with a broad set of features to support diverse use cases. We argue that Manley’s strategy presents plenty of possibilities to be explored for the long travels along European pilgrimage routes and their unknown immediate rural landscapes.

### Wearable guides

Our second chapter of narrative practices in cultural heritage is grouped under wearable guides. All of these use autonomous and multi-sensorial guides in which a deeper connection between digital (audio, textual and/or visual) narratives and the physical space is achieved. We call this integrity between the digital realm of the narrative and the physical world it refers to, which is not only based on context-aware display by GPS tracking and auto-play, but also on literally superimposing some parts of the narrative over the reality by means of state-of-the-art technology: mainly smart glasses and context-aware augmented reality (AR). These wearable guides aim to go beyond the limits of smartphones and tablets in environmental storytelling with new possibilities for presentation, interpretation and engagement with cultural heritage, but there are also many challenges to be faced.

In 2016, a team of researchers from three Greek university institutions joined together to publish the results of the so-called **KnossosAR MAR app** ( Galatis *et al*., 2016). The project aim was to support guided, educational tours in the outdoor archaeological site of Knossos, in Crete (Greece), for secondary school students. For that purpose, they developed and tested a mobile augmented reality (MAR) guide for smartphones and tablets with GPS auto-play.

This guide assists visitors to the archeological site to locate points of interest and it also provides information to help the students gain knowledge about the location through different types of content (textual narratives, audio stories, images and 3D models) ( Galatis *et al*., 2016, 2). Instead of drawing visitors’ eyes to the mobile interface, data is superimposed over the place as if it coexisted with the real world. This allows students to visualize and interact with the archaeological site and its different artifacts in a more direct, intuitive and appealing way.

KnossosAR interface is said to use “the visual metaphor of a radar to display the location of points of interest (represented by dots) relatively to the user’s location and direction”, which is a relevant feature for open-air complexes, where finding a particular spot may be difficult without indications ( Galatis *et al*., 2016, 4). To make this process even easier, they incorporate an alternative means of POI location, which is a dual AR/map view. Students are able to change from the augmentation form to a more familiar map display. In both cases, the dots representing POIs are distinguished with a color code depending on their visibility or occlusion from the observer’s field of view, as the authors address in this project the usual occlusion problem in location-based AR apps for outdoor activities ( Galatis *et al*., 2016, 6). These include situations where a POI may be hidden by a physical obstacle, such as a building and, as a consequence, out of the user’s field of view. On the top of all the previous features, they implemented audio announcements and/or device vibration that the users listen/feel when they approach a POI without the need to be continuously looking at their device ( Galatis *et al*., 2016, 5).

Although this Greek team handle a set of important challenges in enhancing cultural heritage outdoor experience with augmented-reality, they also recognize the need for further improvements to the visitor experience in areas such as: the provision of richer interpretative information and data about the archaeological site as a whole instead of only specific structures, visual clues to show already visited points of interest and alternative ways to show hidden POIs, both in and out of the user field of view ( Galatis *et al*., 2016, 9).

The second case study of wearable guides we include here is TravelAR Slovenia. Developed by the CtrlArt Studio (Ljubljana), the Business Inkubator of Kočevje (Slovenia) and the Business Inkubator of the town of Črnomelj (Slovenia), the project aims can be summarized as follows: to bring the past of cultural heritage back to life through dynamic AR tourism visitor experiences, to create an AR heritage trail that spans sites, towns and cities in Slovenia, and to actively involve local communities in the creation and production of sound narratives for those onsite experiences ( CtrlArt creative studio, 2019).

The project results in two main outputs: the TravelAR Slovenia app and the TravelAR visitor experience. The first one makes use of mobile devices such as smartphones and tablets as a means of communication and interpretation. The second output relies on wearable AR glasses (Epson Moverio BT-350) available at the TravelAR visitor experience locations. These smart glasses are used to supplement the real world of a mobile user with computer-generated virtual content, while also providing visitors with a hands-free approach. In addition, looking through the optical see-through glass offers a more immersive experience than that of mobile devices, since visitors are able to have a better awareness of their context when receiving information. Their visual sense is not displaced from the real world to a mobile device interface, but they are able to see narrative and data as one layer of augmentation on top of the physical world directly through the spectacles they are wearing. In this sense, they get a complete integration of locative narratives with their locations, which may be texts or images, but also 3D artifacts of that place lost over time into the present. To borrow from Hight:

*…cities and the landscape as a whole can now be navigated through layers of information and narrative of what is occurring and has occurred. Narrative, history, and scientific data are a fused landscape, not a digital augmentation, but a multi-layered, deep and malleable resonance of place.* ( Hight, 2006, 2)

The application has great potential for cultural heritage. For instance, in the case of the Žiče Charterhouse, which was a Carthusian monastery in the municipality of Slovenske Konjice in northeastern Slovenia, they created a TravelAR visitor experience to educate, enrich and engage with the standing walls of the past monastic buildings and the heritage site itself ( Kangler & Nadav, 2020). With the Epson Moverio smart glasses, Kangler notes that locals and visitors have the chance to “actually visualize how deep the story is, because of course, your imagination is working better with the vivid pictures from the AR” ( Kangler & Nadav, 2020).

We argue that TravelAR visitor experiences are a materialization of what Hight calls “narrative archaeology” in which technology is not only used to place the artifacts of place lost over time into the present, but also to dig from physical artifacts and find layers of information —pulled from research about lost buildings, eras, people, or events— by the person walking and observing the area with, in this case, wearable devices ( Hight, 2006, 6). A key point of the approach is the use of light smart glasses instead of huge opaque headsets, so the observer is granted a wearable gadget that is pretty close to normal glasses ( Kangler & Nadav, 2020).

A similar approach has been recently proposed by Eran Litvak and Tsvi Kuflik (2020) for the **open-air Hecht Museum in Haifa** (Israel). They developed both a wearable AR guide for smart glasses and a mobile handheld guide for smartphones and tablets. The first makes use of the Everysight Raptor cycling smart glasses, which are said to be the latest technology in cycling wearables. The project aims were to improve the experience of cultural heritage visitors in an outdoor environment, similarly to the two previous projects, and to allow visitors to navigate among selected POIs within the archaeological site while they gain knowledge about them.

Both guides allow visitors to find their way between POIs by following AR marks as well as to verify arrival at a particular location ( Litvak & Kuflik, 2020, 876). The latter is achieved by means of an audio alert of the POI’s name, but also through short-form laying text as one layer of augmentation displayed on top of the physical world while wearing smart glasses. In addition, when a visitor reaches each POI, both guides offer multimedia content to see and/or listen to information about that particular place ( Litvak & Kuflik, 2020, 876). All interactive buttons for audio play, audio progress, AR labeling, and so on, were prepared and tested to facilitate easy navigation and visualization when they are displayed on top of the real environment ( Litvak & Kuflik, 2020, 877–881). Another feature of the application is to spot and label POIs nearby the visitor’s location that are waiting to be discovered ( Litvak & Kuflik, 2020, 876).

Litvak and Kuflik provide visitors of this archaeological site with meaningful information in a noninvasive manner, while they also address a set of challenges of AR applications for outdoor environments, such as: the visualization of content in an augmentation form, the poor visibility of the smart glasses’ display in bright sunlight, the presence of other visitors obscuring views, the inaccuracy of GPS-based location in isolated environments, the ergonomics of smart glasses and the usability of the wearable AR touchless interface ( Litvak & Kuflik, 2020, 875).

Their outputs are described as a prototype or “first attempt to repurpose smart glasses made especially for professional cyclists and built for the outdoors as a platform for the development of mobile museum visitors’ guides for open-air museums” ( Litvak & Kuflik, 2020, 885). Like in both previous case studies, the project focused on outdoor cultural heritage sites. The use of wearable technology that the visitor walks with and observes through may have plenty of possibilities to be applied in the context of pilgrimage routes or, the other way around, we consider that the latter is a perfect context for further research.

### Context-aware games

The third group of narrative practices is classified under the label of context-aware games, that is, playful experiences in which tourists are invited to become players (active visitors/participants) of one game or a series of games bound to one or several locations or entities with cultural, historical or natural interest. The main purpose of context-aware games for cultural heritage storytelling is to enrich tourists’ experiences through a more meaningful player-directed exploration of and interaction with the physical world. They look for a stronger emotional connection between the cultural entity and participants, which is mainly based on turning tourists into active visitors that not only read a place, but also experience the cultural asset through hands-on activities.

As Susan Hazan recalls, while the term audience only covers the acts of listening and watching, an active visitor is encouraged to interact through hands-on, engaging applications. This approach generates novel scenarios for life-long learning, as “whilst we only remember ten percent of what we read, we remember ninety percent of what we do” ( Hazan, 2010, 143).

In the case of the **Raiders of the lost water**, Alessandro Gurrieri with the Ecomuseum Mare Memoria Viva made use of a location-based game to rediscover, raise awareness and enable learning along the coastline of Palermo (Italy), in which currently only 8 km are perceived from 22 km ( Ingrassia & Villodres, 2020, 24–25). In particular, their aim was to show players a set of unknown or hidden access points to the sea that have been slowly stolen from the citizens by urbanization.

For this purpose, they created a playful experience in which participants are divided into teams that ride their bikes from one location to another along a particular open-air coastal area in Palermo. The experience comprises nine phases that take place in nine different sea points. At each location, every team plays a game focused on how the sea was perceived in the past by city inhabitants. Among the activities, teams are asked: to dance at Lido Petrucci, to recognize fish species in photos, to put the lyrics of a historic Italian song about the sea in order, to sing the previous song with ten passers-by, to make sea knots, etc. ^13^ In addition, they have to collect a seawater sample at each location to be poured into a transparent container when they return to the starting point of the tour. In the end, all containers figuratively represent and rejoin the 22 km of Palermo hidden coastline ( Ingrassia & Villodres, 2020, 25).

Raiders of the lost water is a metagame, that is, one that surpasses the rules set for the game, as it is not defined by code, commerce or computation, but by everything “occurring before, after, between, and during the game as well as everything located in, on, around, and beyond the game” ( Boluk & Lemieux, 2017, 11–17). The present case study is a route-specific metagame, which gathers a series of context-aware games and anchors the whole experience in time and space under a unique theme. Raiders of the lost water does not rely on any website, app or related technology that could improve the experience, for instance, on the way from one point to another. They instead created an analogue tangible kit for people who take part in the activity. With a playful approach, this route-specific game is deeply oriented to increase knowledge among participants, reach awareness and foster a critical assessment of the recent transformations of coastal settings.

The next case study is Secret City Trails. Cofounded by Wendy van Leeuwen and Kristina Palavicova in 2016, their aim was to implement the idea of a city discovery game that led local people (and travelers) to new places in their own city instead of continuing to visit the same ones. They created a web platform and app in which users are able to choose and buy a playful walk in more than 50 cities across Europe. Once the selection and purchase are done, participants receive an email with a link to get information about the starting point of the game and the playing times.

Secret City Trails offers a plethora of playful walks. At each stop, players are asked to solve location-based riddles, to learn about curious stories tied to places, to discover hidden landmarks, or to get recommendations for art galleries, free museums and coffee shops, etc. Each walk is created by locals who are paid each time their game is played. This way, they enable participants to discover the city and follow the steps of locals, but with full flexibility regarding timetables. As they are self-guided games, there is no need to adapt the trip to meet a guide in person at a fixed time. In addition, games are instantly bookable, so pre-booking is not required. ^14^ Like in the previous case study, each game is a series of games tied to specific locations within the city. By linking players’ movements in the physical world to their accomplishments in the game, these discoverable walks add a new dimension to in-game storytelling.

The **Escape from the Tower app** is another example of a context-aware game in which the scenery is not an open-air area or urban itinerary, but one iconic building in the city of London, the Tower of London. The independent charity Historic Royal Palaces aimed to engage young people, teenagers and family groups in visiting that British heritage building as well as to enhance their experience, so they decided to develop a location-based game that runs through an app. ^15^ The game invites users to actively participate in a selection of the most famous historical escapes from the Tower of London, in the locations where they happened, while they learn about the historical site ( Reddington, 2010).

Interaction between app users and building heritage was achieved through adding virtual content to the physical site, such as letters, ropes and other virtual material available for players to help prisoners escape. In this experience, app users were not only readers of the immediate physical realm, nor only listeners to an aural narrative tied to the site, but they were also active visitors as they were asked to answer questions and make decisions that affected the game, the overall experience, and let them win prizes ( Reddington, 2010). It is a type of story-game that combines elements of play (gameplay procedures and reward system) with those of stories (characters, setting, and plot) that ensure the engagement of participants with the story while they play the game ( Alexander, 2014, 192–193, 202–203). In this case, there is the added value of location-based activities that are supported by geo-located sensors displayed within the Tower of London. On the whole, we argue that context-aware games represent the possibility for more engaging narratives that may leave visitors/travelers/pilgrims with more meaningful knowledge and memories.

### Simulations

The next group of narrative practices on cultural heritage is focused on 3D recreations of former historical realities that make visible the past and hidden stories attached to events, objects, landscapes, or particular buildings, that is, simulations. All selected experiences are also examples of locative narratives, as 3D data is again triggered by proximity to specific GPS coordinates. Although different kinds of information are integrated to enrich the user experience in the following two case studies, the main focus is on watching and feeling the 3D past space, as a means to enhance the understanding of the contemporary physical realm. Through the simulation of the past, they make visible to human eyes a lost structure and its context or one that has been deeply transformed or even relocated over time.

The first selected case study is **Hidden Florence 3D: San Pier Maggiore App**. It is the result of a collaborative research project led by Fabrizio Nevola (University of Exeter), Donal Cooper (University of Cambridge) and Nicholas Terpstra (University of Toronto), along with The National Gallery (London). ^16^ Their goal was twofold. Firstly, they wanted to make visible the hidden parts of the church of San Pier Maggiore, which was demolished in the 18 ^th^ century, in the city of Florence; that is, in the original setting where houses and shops are placed today. They also aimed to provide an altarpiece from that church, which is today at the National Gallery of London, with a virtual context.

They created a geo-located app that utilizes AR as well as GPS to place the user inside a reconstructed virtual model of the church, both in Florence (native setting) and London (current setting of the altarpiece). The main technical challenge was to integrate “the augmented 3D model on the right location to faithfully recreate the Church of San Pier Maggiore” at both settings. ^17^

The project presents a series of strengths for cultural heritage storytelling such as the spatial, historical and cultural connection created between two different contexts by means of geo-located augmented 3D modeling. This practice could be applied to many other entities that are currently spread, but also bound to cultural itineraries, as a way to recover lost meanings, but also to enrich pilgrimage with invisible connections between distant structures, objects and their contexts. In addition, the application promotes the discovery of the city past by locals and travelers in Florence. In London, it enables museum visitors to enjoy the altarpiece on the original lost context and place, with a sense of space that helps them to better understand the artistic work ( Nevola, 2020). We argue that the San Pier Maggiore App is another example of the “fused landscape” of narrative, history and scientific data envisioned by Hight (2006, 2), with the added value of interweaving distant landscapes in space and time, though still attached to a mobile device interface.

Another good example of narrative simulation is the **Falstad Center V/AR guide**. It was developed by the Falstad center (Falstad-Norway), the Synthetic, Perceptive, Emotive and Cognitive Systems Group (SPECS) at the Barcelona Institute of Science and Technology, the Future Memory Foundation, the ic_ACCESS: Inclusive Strategies for European Conflicted Heritage HERA Project, and Eodyne Systems S.L. in 2018. ^18^

The Falstad Center was a prison camp in Norway and today it is a national center for the education and documentation of the history of imprisonment during the Second World War. They created a tablet-based outdoor AR landscape guide to offer a new way to explore the former prison camp and its context, which are a completely different site today. This approach is based on an active exploration of both the environment and historical material in order to engage students, visitors and educational programs with the memorial site ( Falstad Center, 2019).

As in the previous case study, the technology involved is a geo-located digital reconstruction that is displayed on top of the physical realm along with historical source materials (drawings, photographs, diary fragments, and recorded survivor testimonies) to deliver individualized spatial narratives. Instead of a more traditional approach of passive navigation inside the museum, visitors are led to actively learn and reflect about the whole site by means of exploration through the virtual environment of information ( Falstad Center, 2019). In addition, the appealing and user-friendly interface plays a main role in the discovery and learning process, as it offers the user:

1. A permanent view of the virtual reconstruction and contemporary map of the location of historical source materials (represented by dots) relatively to the user’s location and direction.
2. Alternative means of viewing the virtual environment (ground level or aerial view).
3. Automatic map orientation according to user’s position.
4. Emerging images when user approaches a POI with available information.
5. Full display of buildings’ names when they are closed to the user and hidden functionality for the others ( SPECS UPF, 2018)

As a tablet device is needed for this experience, the app could distract users from the main visiting purpose due to the fact that there is a permanent physical object in between the user and landscape. In addition, the virtual reconstructions of the site structures lack a realistic representation that diminish the perception and interpretation of the historical space. However, it opens the way to an immersive experience of the physical world with several new layers of digital information and narrative about what is happening and has happened, limited only by what it selected in terms of location, types of data, tools and technology.

### Digital exhibitions

The phrase “digital exhibitions” calls to mind a type of spatially organized and visualized expression of thoughts, material and knowledge and to a great extent is based on the display of mixed objects in real space. The adjective “mixed”, in this context, refers to the combinations of physical and digital media in ways that propose new relations between people, space, physical artifacts and digital objects. In other words, the digital exhibitions we are going to analyze add experiential layers of culture to physical space by means of technology that introduces new behavioral codes and allows multilevel spatial exploration.

The first case study we include in the present fifth group of narrative models, with potential to be explored in the production of meaningful content for lesser-known rural museums and heritage sites located nearby pilgrimage routes, is Digital Art Park – NetPark. The project, launched in 2015 by the artistic company Metal, proposes the creation of experiential layers of culture in the public space of Chalkwell Park in Southend on Sea (UK), through digital art works and location-based stories that are connected to the physical space.

The core of the experience is a collection of 14 NetPark apps, of which nine are digital artworks and five are story apps. Each one is related to a particular place or area within the park. By using a mobile device or tablet and headphones, locals and visitors are able to interact with the physical park in a different way, as each piece of work is a new digital layer superimposed on the real landscape that may be “surprising, funny, informative or thought-provoking”. ^19^ For instance, the Matmos App enables users to listen to site-specific music that was created from original sounds from the daily park life. The Mark Grist and MC Mixy App offers poetry tours around the park, while the Spiky Black App is focused on the historic Rose Garden and engages listeners with the exploration of how roses are bred, grown and named, to cite a few examples.

The project is supported by an integrated system of WIFI, physical signage and mono-lights located in the park, as well as the apps and a web gateway. ^20^ Everyone has access to the experience, even if they do not have a device with internet connection. At the same time, the project creators are able to get information about the use of the different experiences. In addition, all NetPark apps were designed for a variety of audiences from young people to adults, but also as shared experiences or individual walks in the park, which stimulates interaction between people and the surrounding space. Users consume audio narratives passively, but the added digital layer to the physical space sometimes activates personal reflection about the topics addressed, sometimes asks for actions and interactions, or sometimes just leads to a passive personal experience in which visitors learn new things while they wander the park.

The next case study is **Writing a haiku...**, developed by Juan Carlos Alonso, José Ángel Parreño and Erik Escoffier from Satellite Studio in 2019. It is a web platform that generates randomized “poems” about places in real time by using a database of coordinate-dependent words in any place of the world, for instance, the one you are standing on ( Brown, 2019).

Writing a Haiku… is inspired in a previous digital exhibition called “Everything every time”, where artist Naho Matsuda created “poetry” from data collected by a variety of sensors located in the city of Newcastle. The resulting random poems were written and displayed in real time. Satellite Studio decided to develop a global version of Matsuda’s project. By using the OpenStreetMap database, they are able to assemble verses based on information from various urban places (names, buildings, streets, bus lines, stops, etc.), but are also related to weather, and local time, for instance. As the short poems are generated with data related to places, the project proposes a funny way to create meaning from that place and to materialize new narratives. We wonder if this fresh idea could also inspire new experiences in which the physical space of the pilgrimage routes and their surroundings are enriched by digital objects randomly created from sensors, onsite databases and pilgrims’ interaction with them (personal memories, components of daily pilgrimage life, places they visited or to visit).

The third case study we include in the group of digital exhibitions is **The Clio**, founded by David Trowbridge in 2012. It aims to “guide the public to thousands of historical and cultural sites throughout the United States”. ^21^ In this project, any physical location with historical or cultural interest around the user, but without a physical marker, is turned into a place of discovery and learning with associated digital data. Members of museums, societies or institutions, as well as students from colleges and universities are invited to contribute to the project by adding information in an open and user-friendly digital platform that also feeds a mobile application ( Trowbridge, 2020). Data is collected in the form of individual entries, which include a short presentation about a site of cultural or historical significance, its location, some images, the backstory and context, as well links to a selection of books, articles, videos, primary sources and credible websites with additional information. In some cases, individual entries are linked together in a walking tour or heritage trail around a topic, area or event. Moreover, there are “time capsule” entries that are thought to allow users to learn about historical events that took place nearby them. ^22^

The Clio is based on the idea that there is history and culture all around us to be discovered and, if we have appreciation for the history and culture that is around us, “we will be leading a richer life with open eyes to a universe that is deeper than we imagined” ( Holbrook *et al*., 2020), but it is also grounded in the consideration that in every single place there is a story to be told and discover, no matter how big or small. The Clio offers a framework to allow the process of creating, telling and discovering stories bound to places in a mixed context that enriches the physical media with digital data and fosters new learning and relationships between people and that place. Although it is currently focused on historical and cultural sites located in the Unites States, it has potential to inspire a global version of the same idea or one focused, for instance, on the thousands of historical and cultural sites along and around European pilgrimages routes.

### Cultural wayfinding

The last group of narrative practices testify the power of new technologies to help people navigate from place to place with graphic communication, visual clues in the built environment, audible information, and tactile elements while they also aggregate or collect cultural data. In this sense, their main focus is to facilitate wayfinding or how users find their way between particular places. In addition, they provide a unidirectional push of information to augment, annotate or add cultural richness to places. They also seek to promote bidirectional narrative exchange between participants and the physical place by directly or indirectly asking questions and collecting their answers or behaviors to generate new narratives, display personalized data or for decision making ( Barber, 2014, 100).

The **Transborder Immigrant Tool** is an example of unidirectional narrative within a wayfinding system. Designed by a group of five artists and writers (Micha Cárdenas, Amy Sara Carrol, Ricardo Domínguez, Elle Mehrmarnd, and Brett Stalbaum) in 2007, their aim was “to guide individuals who were making their way to the US through the deserts of the US/Mexico borderlands to water”. ^23^ The tool offered crucial information about survival as it leads travelers to existing water safety stations during a dangerous journey of several days ( Center for Art and Media Karlsruhe, 2014). In addition, it randomly delivers poetry to those crossing the border in an effort to recover the human side of the border while assisting in their emotional well-being ( Levine, 2014, 152–154). The poems were related to the beauty of the resources of the desert itself.

As it was conceived for humanitarian aid, the app was released as an open-source tool for potential deployment at other border crossings ( Electronic Literature Directory, 2007). It was prepared to allow the mobile phone to receive positional data without internet service. Moreover, the interface is presented as a compass that provides a customized virtual trail and user-based map, and is capable of tracking the spatial coordinates of migrants in real time ( Walsh, 2013, 11). This tool makes hospitality in the complex context of borders possible, an action embedded deeply within the pilgrimage routes’ histories, with the added value of audible poems that turn borders into geo-poetic spaces.

Where the previous tool provides GPS positions and triggers sound files to which the user may react but not respond (unidirectional exchange of information), the next case study encourages the expansion and exploration of the wayfinding system in another way. Run for the first time in Bristol in 2013, **Hello Lamp Post** is an on-street engagement tool designed by PAN Studio that has been already developed for over 25 cities around the world. ^24^ Through a mobile text messaging service (SMS, Whatsapp or Facebook Messenger) or a QR code reader, the project invites people to playfully interact with the urban built environment with a twofold purpose; they aim to provide people with key local information, but they are also interested in gathering important opinions, ideas and perceptions from them that could better inform the development and planning of the city itself ( Ingrassia & Villodres, 2020, 90–91).

Imagine walking through an urban area where you can find any kind of city street features such as a lamp post, a bus shelter, a parking meter, with a mobile phone equipped with any text-message service. Printed signs placed next to those objects or on their surface with sentences such as “Hello, I am a talking telephone box!”, and a reference code or QR code catch people’s attention and invite them to interact. By texting “Hello Telephone Box” and the reference code, or by scanning the QR code, anyone can start a conversation with the urban object that will suddenly “come alive” and ask a series of questions via text message about what the user is doing and how he or she feels about the area. Citizens share their views, concerns, doubts and receive and answer questions while all data is gathered, saved and managed by a web server. This works with a cloud communications platform, that is an artificial intelligence (AI)-driven, conversational system. All collected data is used to feed future conversations between objects and people, but is also shared with local authorities to better inform decision making.

Depending on the needs of the city government, the experience may be implemented to address different concerns regarding wayfinding, culture, tourism, and urban planning. In this sense, up to now there are fewer specific initiatives in the cultural field of Hello Lamp Post than focused on gathering insights for community engagement in urban design, but there are many possibilities to be explored. In addition, Hello Lamp Post has been solely focused on urban settings so far, but what about “waking up” objects outside the city? In particular, we consider that the novel approach envisioned by Hello Lamp Post could be utilized in the context of the pilgrimage routes to promote a connection between travelers and the surrounding environment on a deeper level, but also to acquire and appropriate place-based experiences through interactions between objects or natural entities (the machine) and people (the pilgrims). This way both machine and people could learn from each other about the complex environment of the pilgrimage routes.

The last example we include is **Mnemosyne** ^25^. Developed by the New Media for Cultural Heritage (NEMECH) and the Media Integration and Communication Center (MICC) of the University of Florence, Mnemosyne was first installed and tested in the Museo Nazionale del Bargello in Florence in 2015. The aim was to provide personalized information to museum visitors in real time based on the physical artworks for which the visitor has shown the highest level of interest.

By using detection and re-identification technology in multiple cameras set inside the museum, the system is able to create visitor profiles while they are observing the artworks, without recording information of their facial features, but only on the color and texture of their clothes to capture the most distinctive elements ( Karaman *et al*., 2016, 6–9). Once the visit ends, each visitor is provided with personalized information, which is generated based on an experience-based system (the visitor’s profile) and on a knowledge-based one, but it does not require specific input from users ( Karaman *et al*., 2016, 12–14). All recommended multimedia content is displayed on the touchscreen device placed at the exit of the museum when a particular user approaches it. 

Among the strengths of the system for narrative practices in cultural heritage, we highlight that it is non-intrusive as it does not demand the user to carry any device or to do any action in front of the artwork. Moreover, visitors can navigate through the multimedia content related to the objects for which their interest was higher within the museum, but they can also get information about other related pieces of art in the same museum or in other institutions of Florence, which turns Mnemosyne into a sort of wayfinding system of cultural spaces or entities. However, there is also a physical disconnection between the artwork and the access to the recommended multimedia content as the latter is shown after the visit. In any case, it promotes a third method of narrative exchange where the user unconsciously participates in the generation of personalized multimedia content and this distinctive information is directly delivered to them.

## Conclusions

**Figure 4.**
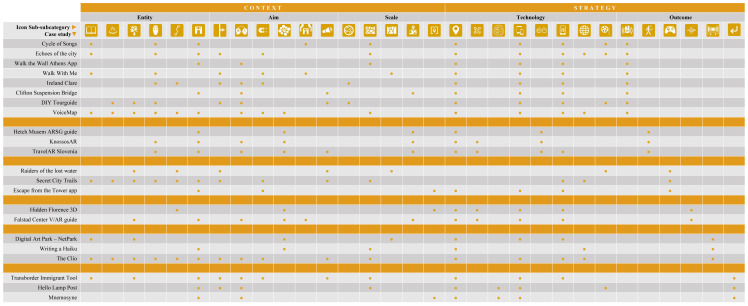
Global analysis of the collection of 22 narratives practices according to categories, subcategories and sub-subcategories presented in Table 1 to Table 6.

The collection of 22 case studies we gather in this review is representative but not comprehensive ( Figure 4). All of them point to narrative practices that disrupt traditional notions of storytelling creation and consumption, as well as foster new types of relationships between people and places of interest. They also demonstrate challenges that are inherent in the act of storytelling, with a particular focus on those that usually constrain the delivery and consumption of content about outdoor cultural sites that are reached on-foot, and movement through space and time.

One rurAllure project aim is to create cohesive narratives about the lesser-known heritage sites of rural areas in the vicinity of four European pilgrimage routes in order to enrich the pilgrims’ on-site experiences. The selected case studies mainly emerged from the exploration of state-of-the-art technologies and the outcomes show how they open up a whole new range of possibilities for discovery and reading about cultural heritage or other entities of particular interest. However, their differences with the older forms of narrative practices are not only related to the instruments used, but the mode of engagement and the resulting experience. When ancient Christians recalled the last days of Jesus’ life on earth by walking and praying along the *Via Dolorosa* in Jerusalem, their prayers gave voice to places and their related stories. This short pilgrimage experience was enriched by adding a sort of oral intangible layer to the physical space, with obvious on-site limitations that grew, even more, when the practice was dis-located and multiplied outside the original setting for centuries. Current technologies overcome some of these limitations; first, by means of tools. For instance, a key feature of narratives displayed through mobile media is that they completely change the relation between content and place, as the former is portable, so it can be moved across the space. However, the real difference with traditional practices is about the experience itself, which introduces significant changes from precedent storytelling.

Selected case studies show that content type impacts the way narrative is told and experienced. Content can be communicated in individual forms such as text and images —common with traditional narratives—, but also audio, animations or videos, or combined in multimedia formats that expand the possibilities of precedent media storytelling in terms of how information is communicated and read. Besides, the experience is also determined by the medium or the way that content is delivered. Today this medium ranges from tangible analogues to mobile devices and wearable gadgets. A 3D model changes the way we learn about former spaces based only on images, for instance, but there is also a huge experiential difference between reading a model on a mobile interface versus using smart glasses versus on a physical printed marker.

With a digital narrative, content is not only site-specific, but it can be tied to physical space and respond dynamically to the place where the user stands. As a consequence, far beyond learning about history, events, architecture, or any other cultural issues surrounding a particular place, we get a deeper sense of the story and the ways that story impacts the meaning, past and present of that place, as Farman highlights. The place or the cultural asset is experienced through various channels and voices that contribute, on the whole, to distinctive and transformative narratives that expand the possibilities of non-digital storytelling. As the rurAllure project proposes, we must explore how these new narrative practices may foster important advancements for the pilgrimage experience in contemporary society and the present review of the broad spectrum of possibilities is a first step.

## Data availability

All data underlying the results are available as part of the article and no additional source data are required.

## Footnotes

^1^ Cycle of the Songs App. Retrieved from: http://www.cycleofsongs.org/resources/app/ [25 January 2021].

^2^ Echoes of the City. Podwalk: the site specific podcast player. What is it?. Retrieved from: http://www.echoesofthecity.com/what-is-podwalk/ [7 January 2021].

^3^ Echoes of the City. Submissions. Retrieved from: http://www.echoesofthecity.com/submissions/ [7 January 2021].

^4^ Dipylon Society for the Study of Ancient Topography. Walk the Wall Athens Project. Retrieved from: http://www.echoesofthecity.com/submissions/ [11 March 2021].

^5^ Studio AB. Walk with me. Retrieved from: http://studioab.co.uk/work/walk-with-me/ [25 March 2021].

^6^ Calvium. Bringing tales of Clifton Suspension Bridge to life. Retrieved from: https://calvium.com/projects/bridge-tales/ [25 January 2021].

^7^ Bridge Tales. Retrieved from: https://play.google.com/store/apps/details?id=com.calvium.suspensionbridge [25 January 2021].

^8^ Discover Ireland. Wild Atlantic Way. Retrieved from: https://www.discoverireland.ie/wild-atlantic-way [11 March 2021].

^9^ Cultural Roadmapp. About us. Retrieved from: https://www.culturalroadmapp.com/about/ [11 March 2021].

^10^ Cultural Roadmapp short video presentation available at: https://www.facebook.com/watch/?v=975108855962556

^11^ Short samples of audio narratives of DIY Tourguide and their presentations are available at: https://www.diytourguide.com.au/audio-tours

^12^ VoiceMap. Create. Retrieve from: https://voicemap.me/ [25 April 2021].

^13^ A short video of the Raiders of the lost water experience is available at: https://www.youtube.com/watch?v=PGvY22jSibo

^14^ Secret City Trails. About us. Retrieved from: https://www.secretcitytrails.com/ [25 April 2021].

^15^ Calvium. Context-aware games at the Tower of London. Retrieved from: https://calvium.com/projects/tower-of-london/ [25 April 2021].

^16^ Hidden Florence. Credits. Retrieved from: https://hiddenflorence.org/about/credits/ [25 January 2021].

^17^ Calvium. Re-imagining a lost icon of Renaissance Florence. Retrieved from: https://calvium.com/projects/hidden-florence-3d/ [25 January 2021].

^18^ A short presentation of the Falstad Center V/AR guide is available at: https://www.futurememoryfoundation.org/

^19^ Metal. NetPark. Retrieved from: http://www.metalculture.com/projects/netpark/ [25 March 2021].

^20^ NetPark. Toolkit. Retrieved from: http://www.netpark.zone/toolkit [25 March 2021].

^21^ The Clio. Retrieved from: https://www.theclio.com/ [2 May 2021].

^22^ The Clio. About. Retrieved from: https://www.theclio.com/about [2 May 2021].

^23^ Net Art Anthology. Transborder Immigrant Tool. Retrieved from: https://anthology.rhizome.org/transborder-immigrant-tool [2 February 2021].

^24^ Hello Lamp Post presentations are available at: https://www.hellolamppost.co.uk/ and http://panstudio.co.uk/project/hello-lamp-post/

^25^ A short presentation of Mnemosyne system is available at: https://www.micc.unifi.it/projects/mnemosyne/
