# Supplementary material for: A collection of narrative practices on cultural heritage with innovative technologies and creative strategies
Source: Open Res Eur. 2021 Oct 25;1:130. [Version 1] doi: 10.12688/openreseurope.14178.1 (PMC10445855; doi:10.12688/openreseurope.14178.1)
Supplement: Peer review history

# Peer review: Reviewer response for version 1

**DOI:** 10.21956/openreseurope.15286.r28076

**Published:** 2021-12-17

**Authors:** Chiara Cecalupo

**Affiliations:** 1 Universidad Carlos III de Madrid, Madrid, Spain

This text offers an extensive description of 22 cases of digital technologies used for actively engaging the public in the promotion of cultural heritage sites. The text is well written and well structured, and is a rich state-of-the art for the project “rurAllure. Promotion of rural museums and heritage sites in the vicinity of European pilgrimage routes”. The project aims to enrich the pilgrims’ experiences *en route* through site-specific storytelling, and this overview serves to develop a deeper knowledge on the good practices already existing around the world.  

 The author uses all these cases to harvest ideas and comparisons for the project rurAllure. Some of them are way more related to the project (i.e. Cultural Roadmapp (Ireland Clare); TravelAR Slovenia), but at the end of the whole overview it is easy to understand the intention of the author behind collecting so many different cases. Pilgrimage routes comprehend many different places, landscapes and buildings, and their promotion needs a holistic expertise.

 The paper can surely become a reference point for anyone interested in digital resources applied to cultural heritage sites. It presents many good practices that connect old activities (such as walking) with new digital experiences. The author has a descriptive and critical approach to cases and always reflects on possible developments and applications. The illustrations are clear, appealing and very well explained. The presence of many web-links is very much appreciated. In some cases, it would also be interesting to know how these projects are planned to be updated. When studying digital practices applied to inhabited places such as cities, one must consider that the human factor is too unpredictable and changeable not to foresee constant updating.

 For future works, I would like to invite the author to reflect also on the impact that pilgrimage had in Europe on the discovery and conservation of cultural heritage from early-Modern era to the 19th century. The production of pilgrimage materials (such as guides or maps) has ancient roots and have always been evolving to meet new needs of the users. How can the Project rurAllure perpetuate this tradition between devotion, movement, and cultural discovery? The author offers a hint in this direction when stating (without offering any bibliography) that “When ancient Christians recalled the last days of Jesus’ life on earth by walking and praying along the Via Dolorosa in Jerusalem, their prayers gave voice to places and their related stories”: this is a wonderful insight that deserves to be the basis of more complex future reflections.

Is the case presented with sufficient detail to be useful for teaching or other practitioners?

Yes

Is the work clearly and accurately presented and does it cite the current literature?

Yes

If applicable, is the statistical analysis and its interpretation appropriate?

Yes

Are all the source data underlying the results available to ensure full access and reuse by other researchers?

Yes

Are the conclusions drawn adequately supported by the data and analysis?

Yes

Is the background of the case’s history and progression described in sufficient detail?

Yes

Reviewer Expertise:

History of Christian Archaeology, History of Christian Collections and museums, Christian cultural heritage

I confirm that I have read this submission and believe that I have an appropriate level of expertise to confirm that it is of an acceptable scientific standard.

# Response

**Published:** 2021-12-21

**Authors:** Estefanía López Salas

**Affiliations:** Universidade da Coruña, Spain

I would like to thank the referee for the time and effort that she has dedicated to provide his report on the paper titled “A collection of narrative practices on cultural heritage with innovative technologies and creative strategies”. I am very grateful to the referee for her positive comments on the paper’s contribution and for her consideration of the collection presented not only as a rich state-of-the-art for the project rurAllure, but also for anyone interested in digital storytelling applied to cultural entities. I also appreciate her understanding of the holistic approach of the present work and her suggestion to develop, in the future, a comparative study between past narrative practices (from early-Modern era to 19 ^th^ century, or even earlier) with recent developments of digital narratives in the pilgrimage context. I completely agree with the referee that this is a field for further exploration that may help us to better understand, examine, and enhance what Jason Farman already recognized as a new form of a centuries-old tradition with the very early example of the Way of the Cross (Farman, 2014, 3–7). This comparative study could lead us to approach how present digital practices should be read within a complex historical development of cultural storytelling in a more critical way. It is especially valuable how the referee expands the perspective on the study even more, providing this new suggestion that will help us to open new paths for further improvement in the future, which I consider is a very helpful, constructive feedback to the author.

# Peer review: Reviewer response for version 1

**DOI:** 10.21956/openreseurope.15286.r27915

**Published:** 2021-11-12

**Authors:** Andreas Vlachidis

**Affiliations:** 1 Department of Information Studies, University College London, London, UK

The paper presents an extensive review of 22 narrative models of cultural heritage applications (digital guides). It reveals best practices, strategies, and state-of-the-art use of technology for enriching visitors’ experience and engagement with cultural heritage.

 The paper is very well-structured and written. The 22 cases studies are summarised under 6 distinct categories, namely; sound-walks, wearable-guides, context-aware games, simulations, digital exhibitions, and cultural wayfinding. Most importantly, the study introduces a well-designed and balanced model and method for systematising the review of cultural heritage applications. The model provides a clear distinction between “context” and “strategy” and enables a review of attributes and characteristics under the categories, entity, aims, scale, technology and outcome, and their specialised sub-categories.

 The value of the model is evident in Figure 4 which provides a comprehensive and succinct view of the review outcome. The only area that the study lacks some clarity is accessibility, both on the level of digital technology and on the level of cultural accessibility of people with disabilities.  It is important that the study discusses how the model can be enhanced to enable reviewing accessibility characteristics of the digital applications as well as how such applications are used to broaden access to cultural heritage sites.

Is the case presented with sufficient detail to be useful for teaching or other practitioners?

Yes

Is the work clearly and accurately presented and does it cite the current literature?

Yes

If applicable, is the statistical analysis and its interpretation appropriate?

Yes

Are all the source data underlying the results available to ensure full access and reuse by other researchers?

Yes

Are the conclusions drawn adequately supported by the data and analysis?

Yes

Is the background of the case’s history and progression described in sufficient detail?

Yes

Reviewer Expertise:

Information Science, Knowledge Bases, Ontologies, Digital Humanities

I confirm that I have read this submission and believe that I have an appropriate level of expertise to confirm that it is of an acceptable scientific standard.

# Response

**Published:** 2021-12-21

**Authors:** Estefanía López Salas

**Affiliations:** Universidade da Coruña, Spain

I would like to thank the referee for the time and effort that he has dedicated to provide his report on the paper titled “A collection of narrative practices on cultural heritage with innovative technologies and creative strategies”. I am very grateful to the referee for his positive comments on the paper’s contribution and his particular consideration of the methodology presented as a “well-designed and balanced model” for systematizing the review of narrative practices on cultural heritage with digital technologies. I also thank the referee for pointing out an issue that the paper did not approach directly: how accessibility of cultural heritage applications could be incorporated into the model to enhance the review of the selected case studies. The referee highlights that the model should consider both accessibility that refers to the extent to which the environment or cultural entity is available and navigable for persons with disabilities, or for persons with other special needs or functional limitations, but also digital accessibility, that is, access to these cultural applications, resources or services. In this sense, first, the model could incorporate a sub-subcategory within the subcategory “Aim” to assess if there is an initial goal to improve the existing accessibility to the cultural entity or not in the context of the action. In addition, another subcategory called “Accessibility” could be added within the category “Strategy” to examine the degree of compliance with accessibility standards and guidelines in the resulting application (Kulkarni, 2019, 92)*. It would be interesting to incorporate and explore this aspect more deeply in a future development of the model.

 * Kulkarni M: Digital accessibility: Challenges and opportunities. *ScienceDirect*. 2019; 31(1): 91-98. Publisher Full Text
